# Unique TCR loci architecture in newts: TRA and TRD separation in *Pleurodeles waltl*

**DOI:** 10.3389/fimmu.2025.1696217

**Published:** 2025-11-28

**Authors:** Magdalena Migalska, Wiesław Babik

**Affiliations:** Institute of Environmental Sciences, Faculty of Biology, Jagiellonian University, Kraków, Poland

**Keywords:** T-cell receptor, Iberian ribbed newt, Urodela, TCR α/β/γ/δ locus annotation, comparative immunology

## Abstract

T cell receptors (TCRs) are central components of the adaptive immune system, yet the genomic organization of TCR-encoding loci is highly complex and has been fully characterized in only a handful of vertebrate taxa. Many important lineages remain largely uncharted, with notable gaps in ectothermic groups. This gap is especially pronounced in amphibians, particularly salamanders (Urodela), where enormous genome sizes have long hindered the availability of high-quality reference sequences. Here, using a recently published chromosome-scale, haplotype-resolved genome of the Iberian ribbed newt, *Pleurodeles waltl*, we report the first comprehensive characterization of all four TCR loci (*TRA*, *TRB*, *TRG*, and *TRD*) in this species. *P. waltl*, alongside the axolotl, is an emerging urodele model, particularly in regenerative biology. Strikingly, we found that the TRA and TRD loci reside on separate chromosomes—a genomic configuration not previously reported in any jawed vertebrate. This unusual organization was confirmed in related species, suggesting it represents an ancestral feature of newts. Moreover, a more recent duplication gave rise to two distinct TRA loci located on two separate chromosomes. Furthermore, using our high-quality genomic TCR reference, we conducted a proof-of-concept TCR repertoire analysis and examined TCR expression through ontogeny in a series of RNA-seq experiments. The genomic toolkit established here paves the way for in-depth studies of TCR diversity in *P. waltl*, strengthening its position as a model species in amphibian immunology.

## 1 Introduction

Immunological research in non-mammalian vertebrates has largely focused on species of commercial importance, such as chickens and several fishes, while amphibians and reptiles remained neglected in comparison. Salamanders (order Urodela), a sister clade to anurans and the second-largest group of extant amphibians, has been particularly understudied (1). While interest in their remarkable regenerative capacities has revealed involvement of both innate and adaptive immunity, the primary focus has been on macrophages and innate immune mechanisms (2). In contrast, adaptive immunity in salamanders remains poorly characterized - a gap of increasing concern as emerging diseases, including that caused by the fungal pathogen *Batrachochytrium salamandrivorans*, threaten populations worldwide (3). Interest in this area is further motivated by long-standing belief about the subdued nature of adaptive immune responses in Urodela (4). Early studies investigating skin graft rejection and mixed lymphocyte reactions in axolotls (*Ambystoma mexicanum*) and various newt species revealed weaker and slower responses compared to those observed in anurans and other tetrapods (5, 6).

Most of the limited knowledge of Urodela adaptive immunity we accrued to date is based on studies in a permanently neotenic axolotl, conducted on long-established laboratory stocks, derived from small, genetically depauperate populations, with wild source populations now nearly extinct (7). However, nowadays, a growing number of salamander species is becoming the subject of investigation (8), facilitating insight into Urodela immunity. Among these, *P. waltl* has emerged as a prominent model (8): large, secondarily fully aquatic species that breeds readily in the laboratory and shares a similar generation time with the axolotl, but naturally undergoes metamorphosis. Yet, most aspects of adaptive immunity in *Pleurodeles* remain poorly characterized. In particular, the composition and diversity of lymphocyte antigen receptor repertoires—such as T-cell receptors (TCRs)—have never been studied in this species. This contrasts with early work conducted in axolotl on TCR repertoires (9–12). Nowadays, a major barrier to such studies in *Pleurodeles* and other urodele species has been the lack of high-quality reference genomes, mostly due to the exceptionally large genome sizes in this group (10–120 Gb, *Pleurodeles* genome is ~20 Gb).

T cells, an essential component of adaptive immunity in all jawed vertebrates, form two conserved lineages: αβ and γδ T cells (13). Their function depends on TCRs, which are heterodimeric molecules composed of either α (TRA) and β (TRB), or γ (TRG) and δ (TRD) chains, respectively. αβ T cells generally recognize peptide antigens presented by major histocompatibility complex (MHC) molecules, and this interaction initiates the adaptive immune response. Depending on factors such as the type of presenting MHC molecule, the cytokine milieu, and the T cell subtype, they can perform various functions—such as killing infected cells, aiding antibody-mediated responses, or stimulating and suppressing inflammation. The functions of γδ T cells are even more diverse and less well defined, with evidence of roles in tissue repair, stress responses, and immune protection against pathogens and cancer. They can bind a wide range of antigens, with an ever-expanding set of ligands, some of which are recognized in the context of MHC or MHC-like molecules; however, unlike αβ T cells, they generally are not restricted by classical antigen presentation rules (14).

Similarly to immunoglobulins, the ligand-binding V domain of each TCR chain is not directly encoded in the germline, but rather assembled during T cell development through somatic recombination of gene segments (13). In general, the TRA and TRG chains assemble their V domains from V and J genes, whereas TRB and TRD chains use V, D, and J genes. In most species studied to date, the TRB and TRG loci are located on separate chromosomes, while the TRD locus is either embedded within TRA or positioned immediately adjacent to it on the same chromosome (15–17). The types of segments used to assemble each chain, as well as the overall genomic architecture of the segmented TCR loci, are broadly conserved across gnathostomes, although species-specific expansions and contractions can alter the number and arrangement of segments. The most striking deviations from this pattern include the complete loss of γδ T cells—and the corresponding TCR chain genes—in squamate reptiles (18), and the emergence of additional TCR chain types, such as the squamate-specific TCRϵ (possibly compensating for the γδ T cell loss) (19, 20), the marsupial TCRμ (21), and the shark NAR-TCR, which features two V domains, one closely related to the V domains of IgNAR (22).

To date, the genomic organization of TCR loci has been resolved only in one urodele amphibian, the axolotl (23). Here, we add annotation for *P. waltl*, using recently published (24), chromosome-scale, haplotype-resolved genome assembly, generated with long-read sequencing and chromosome conformation capture techniques (contig N50s exceeding 20 Mb for each haplotype, scaffold N50 1.2-1.4 Gb). With contiguity improved by four orders of magnitude relative to the earlier assembly available for this species (25), this genome provides a suitable basis for annotating complex immunological loci such as TCRs. Unexpectedly, we found that the TRA and TRD loci are located on separate chromosomes. We examined other newt genomes to confirm that this feature reflects a true lineage-specific characteristic rather than an assembly artifact. Furthermore, we identified two TRA loci on separate chromosomes, likely resulting from a relatively recent and taxon-specific duplication.

To further validate the TCR locus annotations, we analyzed the TCR repertoire of a post-metamorphic individual using an amplicon-based Rep-Seq approach. The availability of fully annotated loci allowed us to apply the state-of-the-art MiXCR pipeline (26) to analyze repertoire diversity. Finally, we investigated the ontogenetic expression of TCR genes through a series of RNA-seq experiments on tissues collected at defined developmental stages (27), confirming expression of all four TCR chains and identifying the onset of adaptive immunity in *P. waltl*.

## 2 Materials and methods

### 2.1 TCR loci annotation in the genome of *Pleurodeles waltl*

#### 2.1.1 Chromosomal location of the TCR loci

First, we identified chromosomes/scaffolds harboring TCR loci in *P. waltl*. To do so, we identified full-length Constant (C) regions of each TCR chain by searching full-length transcriptomes (Iso-Seq) from spleen (SRA accession SRR31583507) with tblastn 2.15 (28) using axolotl TRA (GenBank Acc. AAA98473.1), TRB (AAK11213.1 and AAK11214.1), TRD (AAK33006.1) and human TRG (CAA51165.1) C region protein sequences. We failed to identify any transcripts resembling TCRγ chains in this library; therefore, we further examined assembled *P. waltl* liver RNAseq dataset, and identified a single putative TCRγ transcript. The identified sequences (hereafter referred to as “full-length TCR C sequences”) were used to perform a blastn search against a chromosome-scale, haplotype-phased *P. waltl* genome assembly (v4.0) available through the Max Planck Digital Library (29). We searched and further annotated the reference haplotype aPleWal.hap1, and if needed (see Results) also the other haplotype aPleWal.hap2. The same genome assembly is now also available as NCBI RefSeq GCF_031143425.1 (reference haplotype 1, *aPleWal1.hap1.2022112*). Haplotype 2, *aPleWal1.hap2.20221129*, is available as GenBank assembly GCA_031142525.1. NCBI genome record is accompanied by information on scaffold name correspondence with submitted, Max Planck Digital Library version, as well as detailed quality metrics.

To provide broader genomic context, we also searched the associated annotation file aPleWal.hap1.anno.v1.20230110.gff (and, when needed, aPleWal.hap2.anno.v1.20230110.gff) for known genes usually flanking TCR loci. Additionally, we searched the genomes using blast to check for the possible presence of VHδ genes, i.e., V segments located within the α/δ locus of several non-eutherian species (30), which are much more similar to immunoglobulin heavy chain V sequences than to TCRα/δ V sequences. As queries, we used the following sequences downloaded from the international ImMunoGeneTics information system^®^ IMGT/GENE-DB database (31): Hsapiens_X07448_IGHV1-2*01_V-REGION, Xenopus_Y00380_IGHV1S1*01_V-REGION, and Xenopus_M24677_IGHV5S1*01_V-REGION.

Next, we proceeded with detailed annotation of each TCR locus. We limited the analysis to 2–4 Mb surrounding the location of each TCR C gene, marking the putative core of each locus. To identify putative V and J genes in the vicinity of each C gene, we employed Python-based search algorithm *VJ-gene-finder* v0.2 (32) [https://github.com/simonfrueh/VJ-gene-finder]. Originally, *VJ-gene-finder* was developed to identify and extract functional V and J genes from chicken genomes, based on conserved sequence features that define immunoglobulin V and J genes in many species (such as the presence of conserved cysteine and tryptophan residues in V genes, a Phe-Gly motif in J genes, and presence of appropriate recombination signal sequences (33). However, since these features are broadly conserved among vertebrates (34) the algorithm could readily be applied to search for V and J genes in *Pleurodeles* (with the search for single-exon leader peptides and the default assignment to TRV families disabled). We also modified the downstream annotation pipeline proposed by Früh et al. (32) to account for genes deviating from these canonical patterns and to facilitate detection of exons encoding leader peptides. In particular, we mapped trimmed TCR amplicons (see *Amplicon-based TCR repertoire sequencing* section and **Supplementary Methods** for details) to the corresponding genomic regions. We used amplicon fragments containing the 5′ UTR, leader peptide sequence, and a portion of the V gene to aid in locating leader peptides associated with each V gene identified by the algorithm, as well as to detect V genes that were either pseudogenized (i.e., containing stop codons; these are automatically discarded by *VJ-gene-finder*) or deviating from expected patterns (e.g., length restrictions, etc.).

#### 2.1.2 Manual annotation

The putative V and J gene sequences extracted by the *VJ-gene-finder* were manually curated because the automated search criteria are not entirely specific to the V and J genes (32); see the original publication for details). This means that some of the sequences identified by the algorithm are “false positives” and should be filtered out before the final annotation. The VJ gene assessment involved the following steps: 1) confirmation of global similarity visible in an alignment of all extracted V and J genes for each locus (MAFFT v 7 (35) confirmation of functional Recombination Signal Sequences (RSS) (36), 3) presence of amplicon reads mapping to the V gene and adjacent leader peptide (applied only to V genes). Genes that fulfilled at least two of three of the aforementioned criteria were kept for further consideration.

The final step involved manually annotating each putative V, J, D and C gene in UGENE v. 45.0 (37). We examined each putative V gene for functional features, such as a predicted leader peptide with a start codon, splice donor and acceptor sites, and an RSS using RSSsite, SignalP 6.0 and Spliceator (36, 38, 39). Regions with mapped reads where no V gene was identified by *VJ-gene-finder* were similarly considered, which allowed for the identification of possible pseudogenes (P) and functional genes that deviated from the search criteria defined in the algorithm. Each putative J gene was examined for the presence of a functional RSS and splice site using the aforementioned tools. The final classification of the germline V and J genes as functional (F), open reading frame (ORF), or pseudogenes (P) follows the methodology described by (32) and adheres to IMGT-ONTOLOGY concepts (40). D genes [short stretches of 9–12 bp, flanked by a 5’ RSS with 12bp spacer and a 3’ RSS with 23bp spacer (40)] in TCR β and δ loci were searched for in the vicinity of the last J gene in each C-J gene group, by cross-referencing locations of predicted, functional RSSs. C genes were located by mapping the full-length TCR C sequences (see *Initial location of the TCR loci* section) and visualizing them in the Integrative Genomics Viewer [IGV, v.2.16.2, (41)]. Finally, exons spanning the coding sequence (i.e., without UTRs) of C-GENES were annotated. Exons were denoted EX1, EX2, EX3 and, when applicable, EX4, similar to the descriptions in the gene tables provided at the IMGT database [IMGT/Gene Tables, (42)]. However, we note that only the first exon encodes the extracellular, constant domain, while the rest code for the linker, transmembrane and cytosolic tail of a TCR.

#### 2.1.3 V subgroup assignment, V and J gene numbering, CDR3 delimitation

Locus orientation was established according to IMGT standards based on C-GENE(s) transcription orientation. Following the IMGT nomenclature, TCR V genes with ≥75% nucleotide sequence identity were grouped into V families. The V-GENE family numbering was set arbitrarily, in ascending order from 5’ to 3’ of each locus. Subsequently, within each family, genes were named according to their position relative to the 5’ end of the locus. Pairwise sequence identity was calculated using MatGat (43). J genes were numbered sequentially in ascending order from the 5’ end of the locus, and subgroup numbers corresponded to the J-(D)-C cluster position in the locus (in TRB and TRD loci). We found two separate TRA loci on two different chromosomes/scaffolds (sc3b and sc4a) and named them TRA1 and TRA2, respectively. Some deviations from the standard nomenclature were necessary in the TRD locus (see Results) due to its unusual organization. All identified genes and features (V cassettes with L_PART1, V-REGION and delimitation of V-DOMAIN with signal peptide cleavage site, J genes, D genes, RSSs, splice donors and acceptors, protein coding exons or exon fragments of C-DOMAINs), along with notes on alterations (if applicable), were annotated in GFF files available as **Supplementary Material** associated with this article, with coordinates corresponding to the genome assembly (v4.0) (29). A detailed representation of loci, drawn to scale, was plotted using the *DNA Features Viewer* (v3.1.5). Furthermore, the boundaries of the CDR3 region resulting from the rearrangement of the V and J genes were determined by analyzing the V and J gene sequence alignments. The beginning of the CDR3 region was defined as the first nucleotide of the conserved C104 in the V gene and the end was defined as the last nucleotide of the conserved F in the J gene. Accordingly, the beginning of FR4 was defined as the first nucleotide after the end of the CDR3 region. These anchor points were necessary for a subsequent TCR repertoire analysis (see below).

#### 2.1.4 Validation of TRA locus duplication

Since duplication of entire TCR locus is a rare event, we sought to verify whether this observation might instead represented a misassembly, with haplotigs from divergent TRA haplotypes incorrectly incorporated into the assembly as separate loci. While this scenario was unlikely, given the assembly we analyzed was haplotype-phased, and haplotigs had already been purged by the authors (24), it merited further investigation. To this end, once we had established full extent of the two TRA loci, we performed two additional, genomic analyses. First, we searched for syntenic blocks between the two loci, adding ±1 Mb flanking regions. Clean, continuous correspondence along nearly the entire length of both loci would suggest that one of this regions represents a retained haplotig. Synteny was assessed using ntSynt2, assuming ~1% sequence divergence between assemblies. Second, we examined coverage in and around the two TRA loci after mapping whole-genome sequencing reads. In this analysis, reduction of coverage within TRA loci to approximately half that of surrounding regions would suggest that these loci actually represent two haplotypes of the same locus. We mapped Illumina WGS data from SRA (SRR6001098, SRR6001099, SRR6001109, SRR6001118, SRR6001122, SRR6001123) to the haplotype 1 assembly using bwa-mem2 (44) and calculated coverage in 10 kb windows using bedtools.

### 2.2 Genomic location of TCR loci and TRA duplications in other newt species

Due to the unusual separation of TRA and TRD in *P. waltl*, we wanted to see if this pattern could be found in other newts (that is, salamandrids in the subfamily Pleurodelinae). At the time of analysis, three chromosome-scale genome assemblies generated by the Darwin Tree of Life Project were available: *Lissotriton vulgaris* (GCA_964263255.1), *L. helveticus* (GCA_964261635.1), and *Triturus cristatus* (GCA_964204655.1). To identify chromosomes bearing TCR loci, we performed a BLAST search with protein queries of constant genes of TRA, TRB, TRD, and TRG that were retrieved from the *de novo* assembled adult multi-tissue transcriptomes of these species (27). The analyses were based on primary haplotypes under the accession numbers indicated.

Furthermore, given the apparent presence of two TRA loci in P. waltl, we examined whether a similar pattern of duplication could be detected in other species. In addition to the three species mentioned above, we analyzed transcriptomes from three other newt species reported in (27), which currently lack reference genomes: *Ichthyosaura alpestris*, *T. marmoratus*, and *L. boscai*. Our aim was to identify divergent TRAC genes that might indicate duplication of the TRA locus.

### 2.3 Phylogenetic analysis

To explore the evolutionary relationships of *P. waltl* TCR V genes, we constructed phylogenetic trees using representative sequences from major vertebrate lineages. In addition, we conducted two analyses focusing on constant genes. The first examined TRAC sequences, including TRA1C and TRA2C from the duplicated loci of *P. waltl*, together with TRAC genes derived from transcriptomes of six other newt species (see section 3.2). The second focused on TRBC genes, which appear to have undergone an Urodela-specific expansion (see Results and Discussion), and included genomic sequenced from *P. waltl*, *A. mexicanum*, and several other vertebrates. Details on reference sources and sequences used are provided in the **Supplementary Methods**. Finally, we conducted a phylogenetic analysis of all *P. waltl* TRAV and TRBV genes alongside those of the axolotl—the closest related species with annotated TCR loci (23). Nucleotide sequences were aligned with R package DECIPHER::AlignSeq() (45) separately for TRAV, TRBV, TRDV, TRGV, TRAC and TRBC. The models of nucleotide evolution were identified by ModelTest-NG v. 0.1.7 (46): TVM+I+G4 for TRAV and TRBV and HKY+I+G4 for TRDV, TRGV and TRBC. Maximum likelihood (ML) phylogenies were reconstructed in RAxML-NG 1.2.2 (47), and robustness of the obtained topologies was tested with 100 bootstrap replicates. Finally, we examined the phylogeny of *Pleurodeles* TRAJ and TRBJ genes to highlight their relationships. The short length of the J limits both broader cross-species comparisons and application of complex phylogenetic methods. Therefore the J gene phylogenies were reconstructed from the Jukes-cantor nucleotide distances using BIONJ method (48).

### 2.4 Amplicon-based TCR repertoire analysis

#### 2.4.1 Primer design for Pleurodeles TCR chains

We used an alignment of the identified full length TCR C sequences to design nested primers that where anchored in C genes of each locus (**Supplementary Table 1**). The specificity of primers was confirmed in test reactions performed on early larval sample (prior to the development of adaptive immunity, used as a negative control).

#### 2.4.2 Animals and RNA extraction

The parental animals were obtained from a colony maintained at the Technical University of Dresden (Centre for Regenerative Therapies Dresden, Germany; registration number DD24.1-5131/3346/10) and were subsequently bred at the Jagiellonian University, Kraków, under approval number 13/2023 of the 2^nd^ Local Institutional Animal Care and Use Committee in Kraków [more details in (27)]. For the purpose of this analysis, we collected tissues from one recent metamorph and one early larval stage [stage 38, according to (49)]. Total RNA was extracted from the spleen of the metamorph and from the whole larva (both preserved in RNAlater), using either RNeasy Kit (Qiagen) or RNAzol^®^ RT (Sigma). RNA integrity was assessed using the TapeStation 4150 system with the RNA ScreenTape Analysis Kit, confirming the high quality of both isolates (RIN^e^ values > 9.0).

#### 2.4.3 5’RACE, PCR amplification and sequencing

Amplicon library preparation and TCR repertoire sequencing were based on a quantitative analysis protocol, as described by (50). This method uses 5’ Rapid Amplification of cDNA Ends (5’RACE) and incorporates Unique Molecular Identifiers (UMIs, random DNA sequences that uniquely tag individual cDNA molecules) to correct sequencing errors (51). A separate reaction and library were prepared for each TCR chain. Briefly, (details in **Supplementary Methods**), 250 ng of total RNA was reverse-transcribed using 5′RACE with SMARTScribe(TM) Reverse Transcriptase kit (TaKaRA), custom template-switch oligos containing UMIs, and a chain-specific reverse primer located in a C gene. Two PCR reactions followed (with Q5^®^ High-Fidelity 2X Master Mix, NEB), resulting in uniquely tagged, sequencing-ready libraries. The amplicons were sequenced on an Illumina NextSeq (paired-end, 2×300 bp cycles) as a part of a larger sequencing run.

#### 2.4.4 TCR repertoire analysis

We analyzed TCR repertoires using MiXCR (v4.7.0; (26)), following quality control with FastQC (52). Despite high quality of reads, initial analysis showed varying amount of non-target reads (particularly for TRG, likely caused by low expression levels; see Results). To mitigate the effect of non-target reads, we filtered the reads prior to analysis using a BLAST-based approach (initial and post-filtering sequencing depths for each amplicon are provided in **Supplementary Table 2**). Next, custom reference libraries were created for each TCR locus, adding manually curated features for CDR3 delimitation (where automatic detection failed). For TRD, we used the haplotype 2 annotation, which, based on IsoSeq mapping results, likely better reflected the locus structure. All used sequences and the updated custom MiXCR libraries are available in OSF repository osf.io/f6kez. Repertoires of each chain were analyzed separately, subsampled to 400,000 reads, and clonotype characteristics were visualized in R (v4.0.2) (53). Details of the analysis are in **Supplementary Methods**.

### 2.5 Ontogenetic expression profile of TCR genes

Expression level of TCR loci across tissues and throughout ontogeny was analyzed using RNAseq data from Babik et al. (27) – the list of samples together with accession numbers is in **Supplementary Table 3**. RNAseq reads were mapped to genomes with hisat2 (54) and reads mapped to C genes of each TCR locus (as annotated in this work) were counted with featureCounts v. 2.0.8 (55). Expression was estimated as Fragments Per Kilobase of transcript length per Million reads mapped (FPKM). Subsequently, when multiple C genes were present per chain type (e.g., six C genes in case of TRB), FPKM were summed to reflect overall chain expression in a given sample.

## 3 Results

### 3.1 Genomic location and organization of TCR loci in *Pleurodeles waltl*

#### 3.1.1 TRA

**Figure 1:**
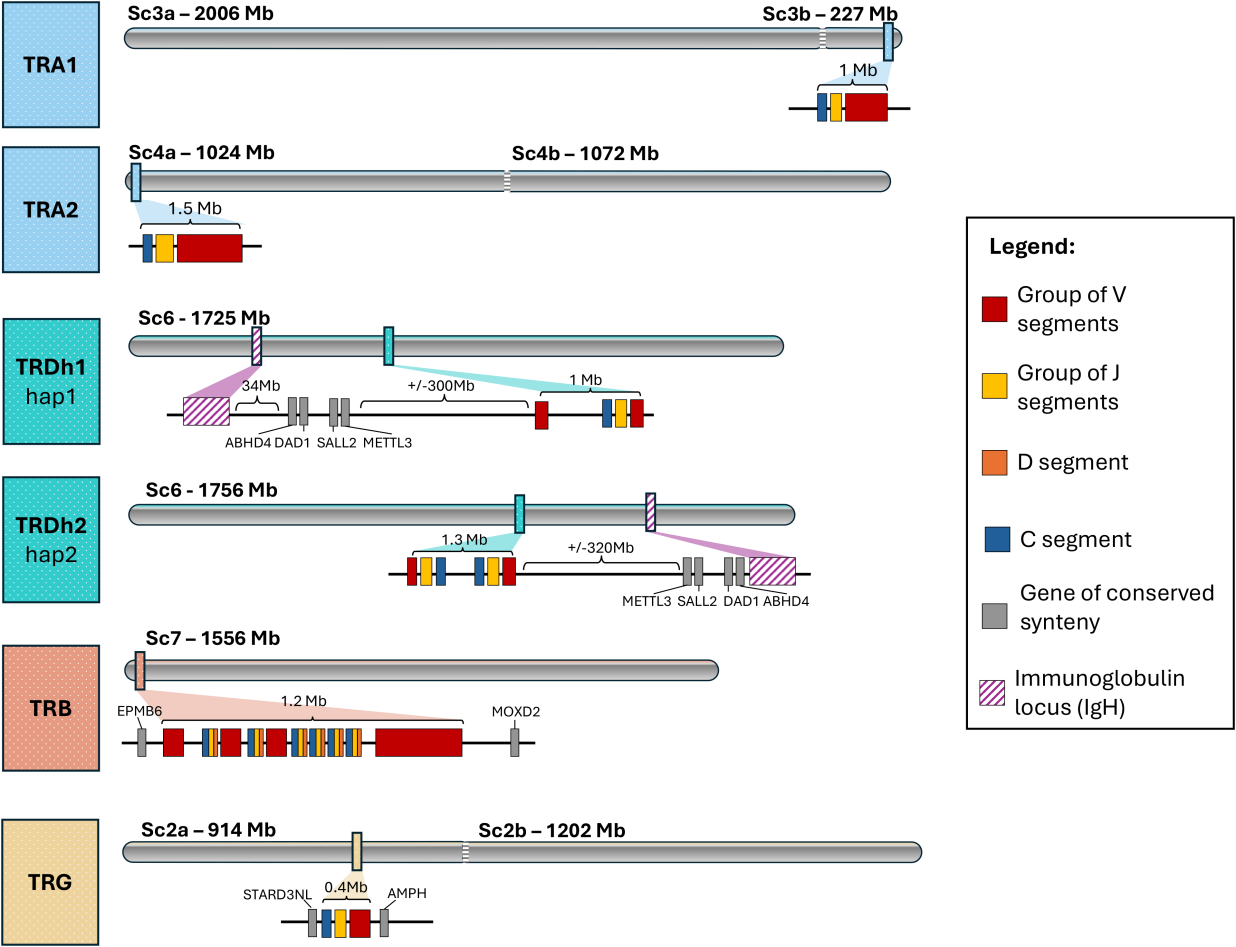
Location and organization of TCR loci in *Pleurodeles waltl*. This schematic (not to scale) shows the overall chromosomal positions and gene organization. Legend for gene groups is provided. “Sc” = scaffold; the “a” and “b” suffixes indicate scaffold splits in the assembly introduced by the original authors to comply with database sequence-size limits; these do not necessarily correspond to centromere positions.

**Figure 2:**
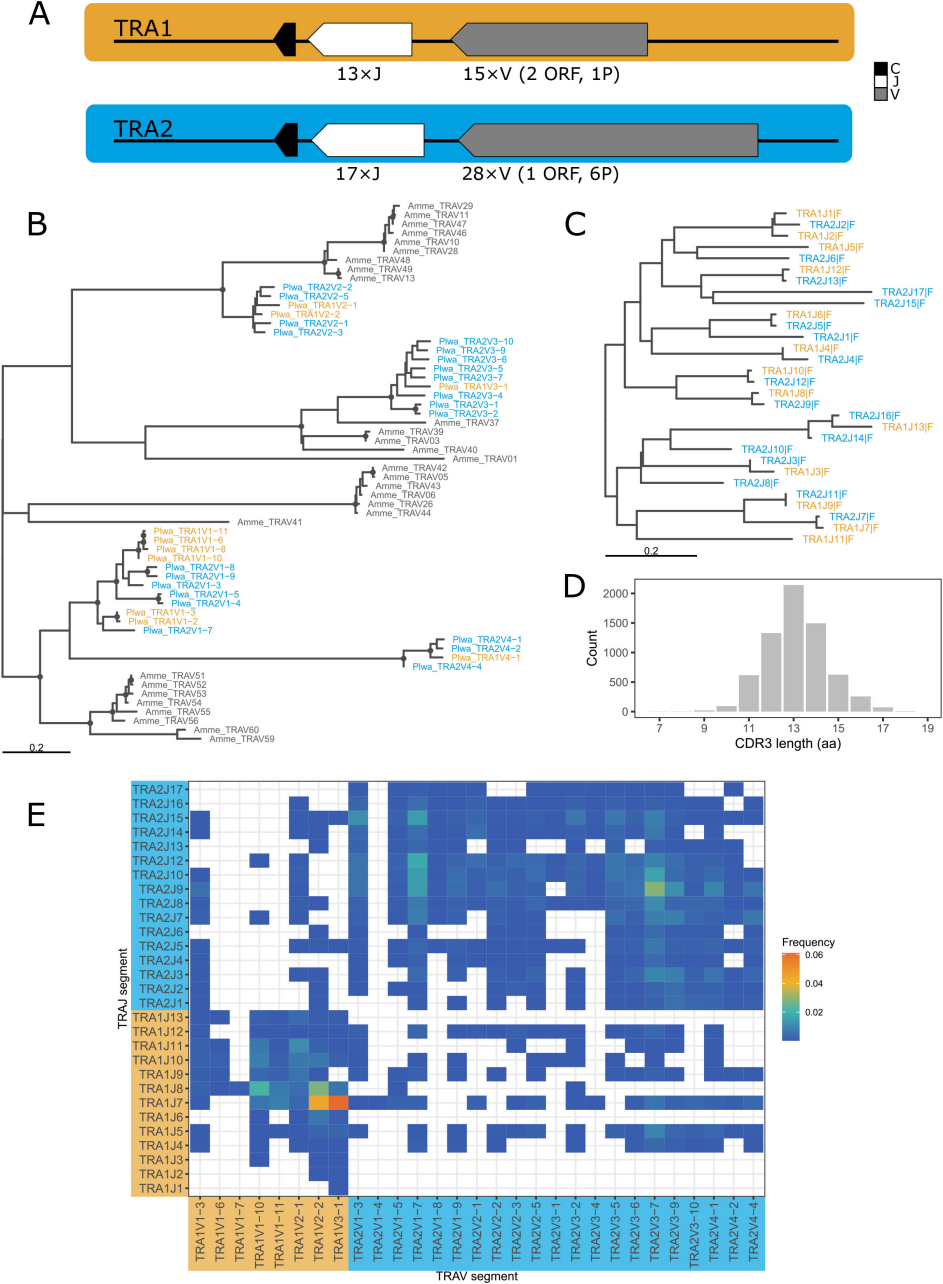
TRA V and J gene phylogeny and splenic repertoire characteristics. **(A)** Schematic (not to scale) of the two TRA loci in *P. waltl*, with transcriptional orientation indicated by arrows. Two chromosomes are shown in different colors; this color-coding is retained in panels **(B, C, E)** to indicate chromosome of origin. **(B)** ML tree of functional *P. waltl* TRAV genes with axolotl TRAV genes in grey. **(C)** BIONJ tree of *P. waltl* TRAJ genes. Trees in B and C are midpoint-rooted. Dots indicate clades with bootstrap support of min. 70%. **(D)** CDR3 spectratype showing distribution of CDR3 lengths (in amino acids), weighted by clonotype counts. **(E)** Grid plot of TRAV–J pairing frequencies (heat-map scale).

Initial IsoSeq screening revealed two highly similar TRA C chains (96% nucleotide identity in the coding region). Mapping to the genome placed them at two separate locations on different chromosomes (scaffolds sc3b and sc4a), each accompanied by a distinct set of J and V genes. Subsequently, we designated them TRA1 (sc3b) and TRA2 (sc4a), using these names as locus descriptors in subsequent gene naming. Four V gene families (groups of V genes with ≥75% nucleotide identity) were defined globally, that is, regardless of chromosomal location. Both TRA loci display a translocon structure, each comprising a single C gene, followed by J and V gene clusters (**Figures 1**, **2A**, **Table 1**, detailed map in **Supplementary Figure 1**). All of the genes were oriented in the opposite direction with respect to the scaffold, prompting the designation of the locus as “reverse” [REV; according to IMGT^®^ ‘locus orientation’ concepts (40)].

**Table 1:** Summary of TCR loci location and numbers of genes identified in *Pleurodeles waltl*.

Locus	Scaffold	Start	End	Number of segments identified							other
				V			J			C	
				F	ORF	P	F	ORF	P	F	
TRA1	3b	220,001,322	221,009,097	12	2	1	13	0	0	1	
TRA2	4a	1,329,156	2,849,870	21	1	6	17	0	0	1	
TRB	7	17,897,156	19,122,406	24	6	2	14	7	0	6	6× D gene
TRDh1	6	682,022,177	683,096,936	3	1	0	1	0	0	1	
TRDh2	6	1,043,651,231	1,044,968,827	3	0	1	3	0	0	2	1× JV germline
TRG	2a	611,944,613	612,337,084	3	1	0	2	0	0	1	

V – variable; J – joining; D- diversity; F – functional; ORF – open reading frame; P – pseudogene.

We further performed additional genomic analyses to exclude the possibility that one of the TRA loci represented an assembly artifact, such as an unpurged haplotig. Synteny analysis revealed limited regions of similarity (**Supplementary Figure 2**), primarily encompassing TRAC and TRAJ genes, along with smaller segments overlapping certain TRAV genes. These shared regions did not extend across the full loci and are more consistent with genuine duplication followed by subsequent sequence divergence in less constrained regions. Read coverage across the TRA loci showed no evident deviations from the neighboring genomic context (i.e., flanking regions up to 10 Mb), particularly no consistent reduction in read depth (**Supplementary Figure 3**). Collectively, given these results, as well as the fact that the assembly itself was generated using long-read sequencing and purged of haplotigs (24), it seems highly unlikely that either TRA locus is an assembly artifact. However, we note that, based on genomic data alone, it is not possible to unequivocally exclude alternative explanations. Molecular cytogenetic approaches, such as fluorescence *in situ* hybridization (FISH), would provide definitive confirmation of the locus duplication; however, such analyses are beyond the scope of the present study.

Notably, neither TRA locus was associated with TRD genes or with conserved syntenic genes typically flanking the TRA/D locus [found in e.g., coelacanth, *Xenopus* and many amniotes, (30, 56)], such as *METTL3* (methyltransferase-like 3), *SALL2* (spalt-like transcription factor 2), *DAD1* (defender against cell death 1), and *ABHD4* (abhydrolase domain-containing protein 4).

#### 3.1.2 TRD

Screening of IsoSeq data suggested the presence of two TRD C genes differing in their 3′UTRs. However, mapping these reads to the haplotype 1 of the genome assembly (Hap1) identified only a single TRDC gene on chromosome 6, with the second copy notably absent. A search of the alternative haplotype (Hap2) revealed two putative TRD genes, located adjacently in inverted orientation on chromosome 6 (**Figure 1**). Regardless of structural differences between haplotypes (which may, to some extent, reflect assembly artefacts), the TRD locus is always located on a separate chromosome from the two TRA loci (**Figure 1**). While we present both genomic organizations, combined evidence from expression data (IsoSeq and repertoire sequencing) suggests that the Hap2 structure is more accurate. We note, however, that TRD structure is likely not fully resolved in either haplotype.

The TRD locus on haplotype 1 (hereafter TRDh1) consists of a single TRDC–J–V gene cluster, with an additional set of V genes in the opposite orientation. In contrast, haplotype 2 (TRDh2) contains two TRDC–J–V clusters arranged in opposite orientations (**Figure 1**, detailed maps in **Supplementary Figure 4**; correspondence between haplotype’s genes **Supplementary Figure 5**, gene counts in **Table 1**). TRDh1 spans ~1 Mb, while TRDh2 extends over 1.3 Mb. This length difference arises mostly from the absence in TRDh1 of the second, full TRDC–J–V cluster present at the 3′ end of TRDh2 (**Supplementary Figure 5**). V genes were classified in three families. Both haplotypes contain atypical V gene arrangements, confirmed by mapping TRD amplicon and IsoSeq reads to the genome. In TRDh1, one V gene is associated with two upstream L-PART1 exons, while in both haplotypes, some L-PART1 exons appear shared between two distinct V gene. Additionally, TRDh2 includes a germline-encoded, pre-joined V region with a fixed V–J rearrangement. Moreover, we identified a non-canonical J gene (TRDh2J2-2), in which the otherwise conserved FGxG(T) motif is replaced by a FKRV motif. Notably, a similar substitution, involving two positively charged amino acids, was observed in the axolotl (FKKGS in TRDJ1 (57). Finally, no D genes were identified in either haplotype, despite using an approach that successfully detected D genes in the TRB locus, which strongly suggests the absence of D genes in *P. waltl* TRD locus.

We also found no evidence of VHδ genes associated within either version of TRD locus. A BLAST search identified a gene cluster (~14 Mb) with 35–65% amino acid similarity to VH genes from human and *Xenopus laevis*, located ~320 Mb upstream (Hap1) or ~323 Mb downstream (Hap2) of the TRD locus (**Figure 1**). These genes likely represent a bona fide immunoglobulin locus, whose characterization is beyond the scope of this study.

Syntenic genes typically flanking the TRA/D loci in many species (*ABHD4*, *DAD1*, *SALL2*, *METTL3*) were found ~300 Mb from the TRD locus, with <1 Mb between *DAD1* and *SALL2*, and no TCR genes (TRA or TRD) in between (**Figure 1**; **Supplementary Figure 6**). In Hap1, this syntenic block lies ~300 Mb upstream of the TRD locus and ~34 Mb from the putative IgH locus. In Hap2, it is ~320 Mb downstream, directly adjacent to the IgH locus.

#### 3.1.3 TRB

Initial screening suggested the presence of six TRB C chains, all located near the beginning of scaffold 7. Five showed 55–64% amino acid identity with TRBC 001–004 (23), and with TRBC1–3 from the pre-genomic axolotl description (9, 11). The sixth, more divergent chain shared 65% identity with axolotl TRBC 005 (23), and TRBC4 in (11).

**Figure 3:**
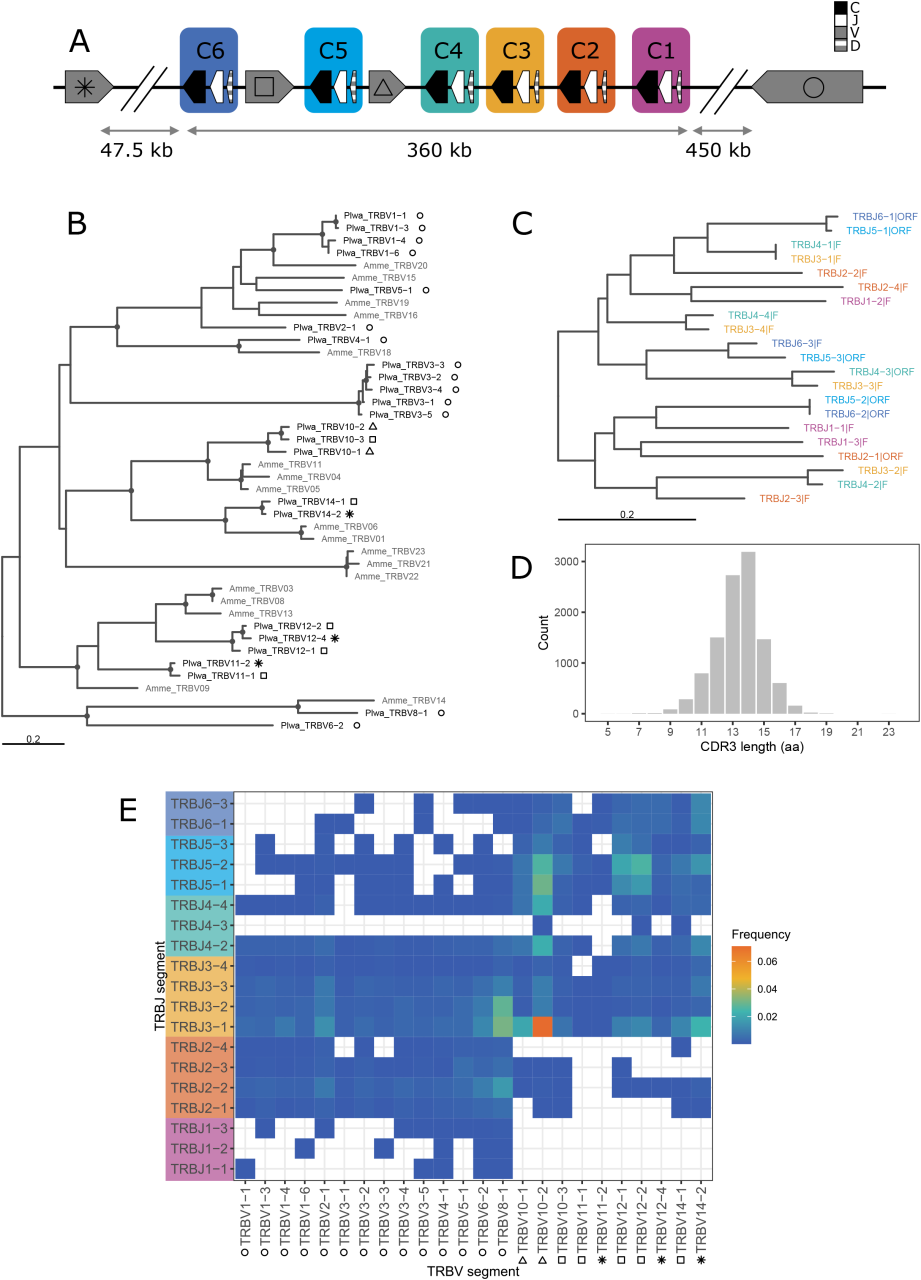
TRB gene phylogeny and splenic repertoire characteristics. **(A)** Schematic (not to scale) of the TRB loci in *P. waltl*. TRBC–J–D clusters are color-coded, V gene groups (in grey) are marked with distinct symbols (circle, square, triangle, asterisk). These colors (TRBJ) and symbols (TRBV) are also used in panels **(B, C, E)**. **(B)** ML tree of functional *P. waltl* TRBV genes with axolotl TRBV genes in grey. **(C)** BIONJ tree of *P. waltl* TRBJ genes. Trees in B and C are midpoint-rooted. Dots indicate clades with bootstrap support of min. 70%. **(D)** CDR3 spectratype showing CDR3 length (in amino acids) distribution, weighted by clonotype counts. **(E)** Grid plot of TRBV–J pairing frequencies (heat-map scale).

The TRB locus exhibits a complex architecture, comprising six TRBC–J–D units, three smaller inverted TRBV clusters (two interspersed among the TRBC–J–D units), and a large, more distant V gene cluster located ~450 kb away, in the same transcriptional orientation as the TRBC–J–D units (**Figure 1**, **Figure 3A**, detailed map in **Supplementary Figure 7**; **Table 1**). This distant V cluster is separated from the main locus by a group of trypsin genes (annotated as *TRY2* = *PRSS2* or *TRYP* in the genome annotation). The TRBC–J–D units were numbered according to locus orientation (REV), which is opposite to the scaffold orientation. We identified 14 V gene families, with members of families 1–8 located solely in the distant cluster, and members of families 9–14 spread in the three smaller, inverted clusters (**Supplementary Figure 7**).

The entire TRB locus, spanning from the first to the last V gene, covered 1.2 Mb. It was flanked on one side by two genes annotated as *TRPV6* (Transient Receptor Potential Cation Channel Subfamily V Member 6) and *EPHB5* (ephrin type-B receptor 5). The latter (or related *EPHB6*) has been reported in this position in several amniote species (16, 58). On the opposite side of the locus (downstream on the scaffold, but upstream relative to the IMGT 5′ locus orientation), the nearest gene was annotated as *CLEC2L* (C-type lectin domain family 2 member L). *MOXD2*, a gene that flanks the 5′ end of the TRB locus in many amniotes (15, 16, 58), was located ~24 Mb further downstream on the scaffold.

#### 3.1.4 TRG

RNA-seq data indicated the presence of a single TRG C gene, which mapped to scaffold 2a. The locus displayed a simple translocon organization, comprising a single TRGC gene, two J genes, and four V genes belonging to three families (**Figure 1**, detailed map in **Supplementary Figure 8**; **Table 1**), all in REV IMGT orientation. The locus is flanked by genes conserved across amniotes (58, 59): *AMPH* (amphiphysin) at the IMGT 5′-borne end (corresponding to the 3′ end of the scaffold), and *STARD3NL* (= *MENTO*; STARD3 N-terminal-like protein) at the opposite end.

### 3.2 Separation of TRA and TRD loci in newts

In all examined genomes, the TRA and TRD loci were separated onto different chromosomes. In *L. vulgaris* TRA was located on CHR_2_1, TRD on CHR_5. *L. helveticus* it was CHR_3_1 and CHR_5_1, respectively. In *T. cristatus* there is evidence for a duplicated TRA locus on two separate chromosomes, CHR_3_2 and CHR_8, while TRD is on CHR_1_2. In all cases we were also able to identify location of putative TRB and TRG loci, each located on a separate chromosome (from each other, and from both TRA and TRD).

### 3.3 Phylogenetic analysis

#### 3.3.1 TRA

**Figure 4:**
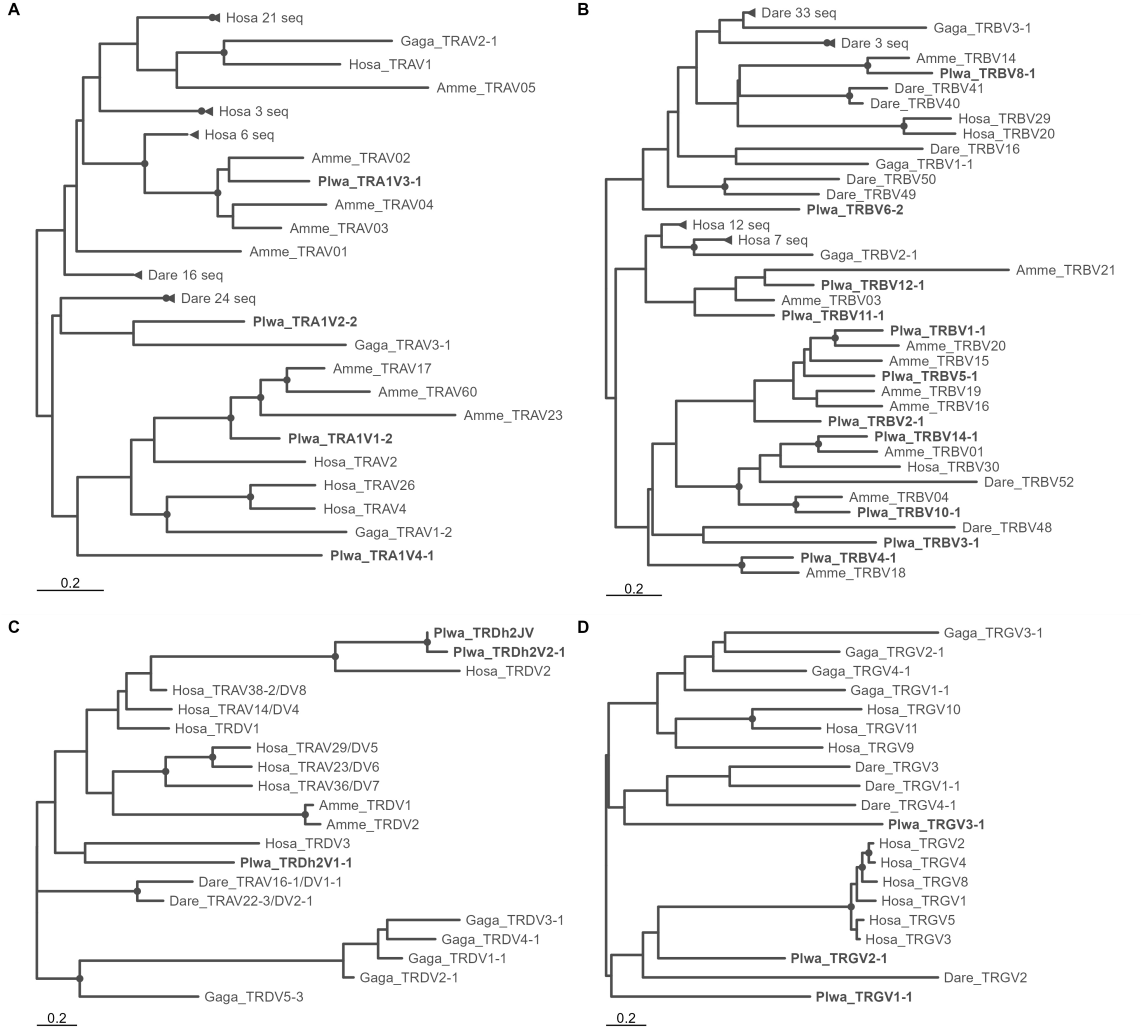
Maximum Likelihood phylogenies of TRAV, TRBV, TRDV and TRGV gene family representatives from various vertebrates. **(A)** TRAV, **(B)** TRBV, **(C)** TRDV, **(D)** TRGV. Trees are midpoint-rooted. For *P. waltl*, only representatives of TRBV families containing putative functional genes are shown. In **(A, B)** clades containing only sequences from a single species are collapsed and shown with triangles at a branch tip, with species and number of sequences collapsed indicated. Species abbreviations: Hosa – *Homo sapiens*; Gaga – *Gallus gallus*; Amme – *Ambystoma mexicanum*; Plwa – *Pleurodeles waltl*; Dare – *Danio rerio*. Dots indicate clades with bootstrap support of min. 70%.

Phylogenetic analysis of representatives from all TRAV families across several vertebrate species shows that the four *P. waltl* TRAV families are highly divergent and likely predate the origin of bony fishes (**Figure 4A**). Only two *P. waltl* TRAV families cluster with axolotl sequences, while two axolotl families lack *P. waltl* counterparts, indicating differential loss of TRAV genes in salamander lineages. Several lineage-specific expansions are evident in salamanders (**Figure 2B**), and each expanded family in *P. waltl* is represented at both TRA loci. The phylogeny of TRAJ genes reveals a striking pattern of multiple, highly similar sequence pairs located at the two TRA loci (**Figure 2C**), further supporting the recent origin of TRA1 and TRA2. However, the presence of longer branches containing representatives from only a single locus suggests some degree of locus-specific gene loss.

The only other newt species for which we obtained genomic evidence of TRA locus duplication was *T. cristatus* (see above). Transcriptome analyses of three additional newt species indicated that only its sister species, *T. marmoratus*, may also carry a similar duplication (or at least two divergent TRAC genes; without a genome assembly, their chromosomal positions cannot be determined). Phylogenetic analysis of the constant genes (**Supplementary Figure 9**) showed that the two TRAC genes in *P. waltl* are not orthologous to the duplicated genes in *Triturus*. In contrast, the duplicated sequences identified in the two *Triturus* species form matching, orthologous pairs. This suggests that this is a distinct, *Triturus*-specific duplication, that either arose within the *Triturus* lineage (with one copy rapidly diversifying) or was retained only in this clade, as one copy follows species phylogeny while the other forms a distinct, *Triturus*-specific clade.

#### 3.3.2 TRB

The phylogeny of *P. waltl* TRBV families, in the context of sequences from other vertebrates, reveals both ancient divergences, likely tracing back to the base of bony fishes, and more recently diverged families (**Figure 4B**). As with TRAV, some *P. waltl* families cluster with axolotl sequences, while others are specific to *Pleurodeles* (**Figure 3B**). Two *Pleurodeles*-specific expansions account for the relatively large size of the distal V unit (rightmost in **Figure 3A**, designated with a circle). Genes from this cluster group with axolotl counterparts from an analogous genomic location [Vβ14–23; “cluster B” in (23)]. Similarly, genes from inverted clusters group with likewise oriented axolotl genes [Vβ1–13; “cluster A” in (23)]. The phylogeny of TRBJ sequences suggests block duplications of entire TRBC–J–D units, with units C3–C4 and C5–C6 forming closely related pairs, while the C1 unit TRBJ1 sequences are markedly different from all others (**Figure 3C**). The divergent *P. waltl* TRBC1 gene (located closest to the locus 5′ end but near the scaffold 3′ end, **Figure 3A**) clusters with axolotl TRBC 005, which is similarly positioned [last of that species’ TRC–J–D gene clusters, (23)]. In contrast, the TRBC2–6 cluster forms a sister clade to axolotl TRBC 001–004, each representing a species-specific expansions (**Supplementary Figure 10**).

#### 3.3.3 TRD and TRG

*P. waltl* exhibits low diversity of TRDV and TRGV genes, although the sequences themselves are highly divergent (**Figures 4C, D**). The phylogenetic relationships are poorly resolved, and both TRD and TRG V genes appear to evolve faster than TRA and TRB genes (as indicated by the scale differences between **Figures 4A-D**). TRDV sequences from *A. mexicanum* show little similarity to those of *P. waltl*.

### 3.4 TCR repertoires

This analysis was primarily intended as a proof of concept and a quality check for the reference we provide, rather than a comprehensive assessment of TCR diversity in *P. waltl*. As such, we report repertoire sequencing data from a single individual, and emphasize that these results may not reflect species-wide patterns.

The majority of TRA V–J pairings occurred within loci: 28.4% of clonotypes used TRA1V–TRA1J, and 61.1% used TRA2V–TRA2J. Apparent cross-locus pairings (TRA1V–TRA2J or TRA2V–TRA1J) accounted for approximately 10% of clonotypes. Gene usage was clearly biased, with the most frequent combination (TRA1V3–1 and TRA1J7) encountered in ~ 6% of all clonotypes (**Figure 2E**), and could not be explained by positional proximity (**Supplementary Figure 1**).

TRB V–J usage also shows clear bias, only partially attributable to genomic location (see **Supplementary Figure 7**). For instance, TRBV8-1, one of the most frequently used genes, is located at the end of the large cluster of V genes sharing the same transcriptional orientation as the TRBC–J–D units (denoted with a circle in scheme **Figure 3A**). It lies closest to the TRBC–J–D clusters, yet is still nearly 0.5 Mb away. Conversely, the most common pairing—TRBV10–2 with TRBJ3-1—does not have an obvious positional rationale (**Supplementary Figure 7**). All six TRBC–J–D clusters are utilized, but unevenly. TRBJ1 and TRBJ2 primarily pair with V genes from the large, distal V cluster, while TRBJ4, TRBJ5, and TRBJ6 are more often associated with the inverted V genes (marked with a triangle, square, and asterisk in **Figure 3E**). TRBJ3 genes are the most frequently used overall, appearing in rearrangements with both standard and inverted TRBV genes.

**Figure 5:**
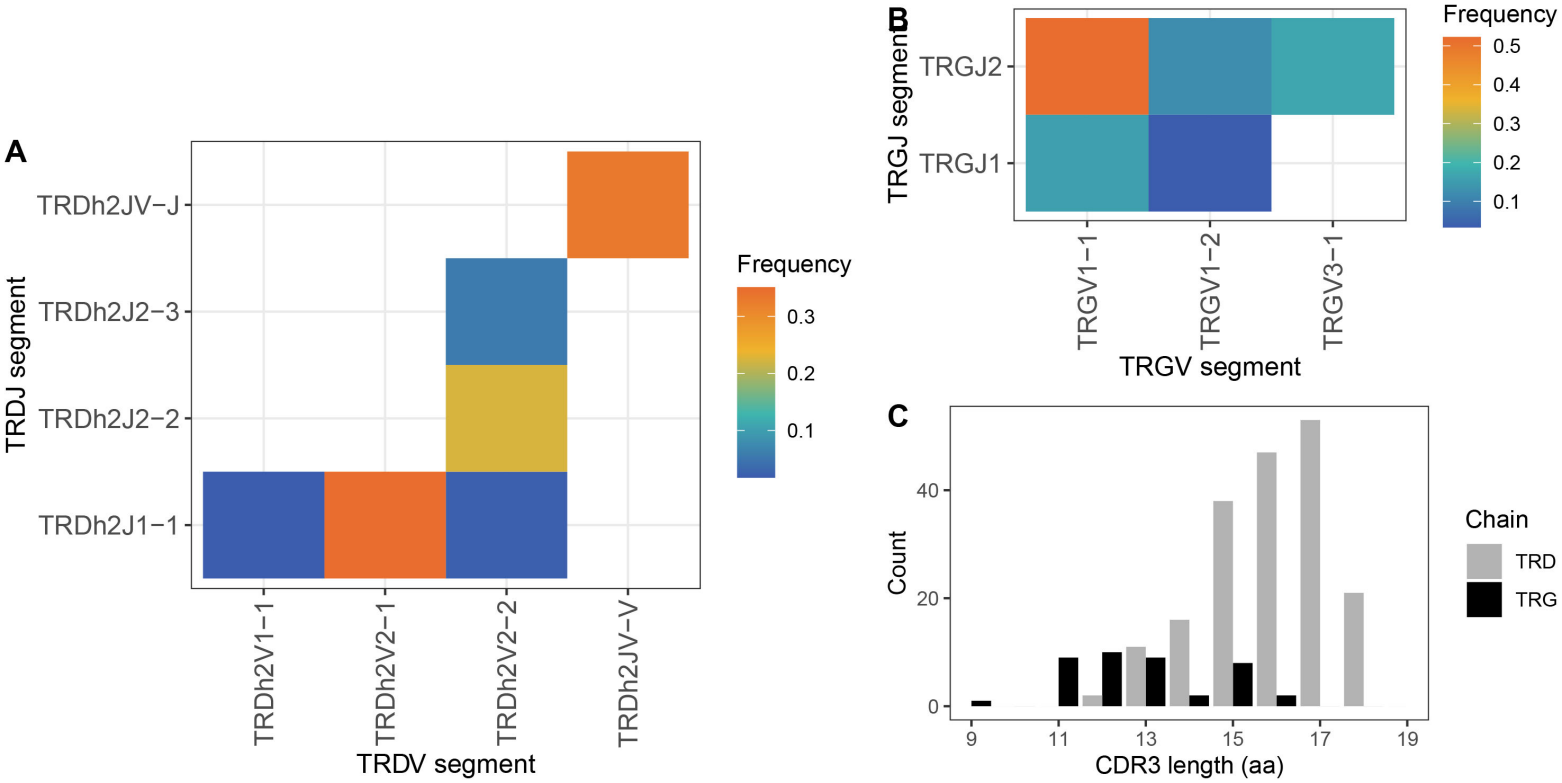
TCR γ and δ splenic repertoire characteristics. **(A)** Grid plot of TRDV–J pairing frequencies (heat-map scale). **(B)** Grid plot of TRGV–J pairing frequencies (heat-map scale). **(C)** CDR3 spectratypes showing amino acid length distributions weighted by clonotype counts; black bars = TRG chains, grey bars = TRD chains.

The TRD repertoire also showed preferred combinations (**Figure 5A**). As expected, the germline-joined V–J gene was found exclusively as a single, fixed combination. Moreover, this was the second most frequent combination, found in one-third of TRD clonotypes.

The TRG repertoire was limited and showed a strong bias, with a single pairing: TRGJ2 and TRGV1–1 accounting for over 50% of all clonotypes (**Figure 5B**). Notably, these two genes are positioned at opposite ends of the TRG locus (**Supplementary Figure 8**). However, this result should be interpreted with caution. Due to the very low expression levels of TRG in spleen (see below), generating reliable amplicons was challenging, and the observed repertoire may not accurately reflect the true diversity.

Clonotype diversity in the sample (from spleen of a recently metamorphosed individual) was 4,207 for TRA, 8,869 for TRB, 134 for TRD, and 49 for TRG (additional diversity metrics are provided in **Supplementary Table 2**). In silico CDR3 spectratyping revealed typical bell-shaped distributions for TRA (**Figure 2D**) and TRB (**Figure 3D**), with mean CDR3 lengths of 13.1 and 13.4 amino acids, respectively. The TRG repertoire showed a flatter distribution with a mean CDR3 length of 12.4 amino acids (**Figure 5C**), though these metrics are likely unreliable given the small number of recovered, valid clonotypes. TRD CDR3s, by contrast, were longer and showed a highly skewed length distribution, with a mean length of 14.8 amino acids (**Figure 5C**). Major TRD CDR3 sequences were easily attributable to germline V and J genes (with some additional nucleotides), but without putative D genes.

### 3.5 Ontogenetic expression profile of TCR genes

**Figure 6:**
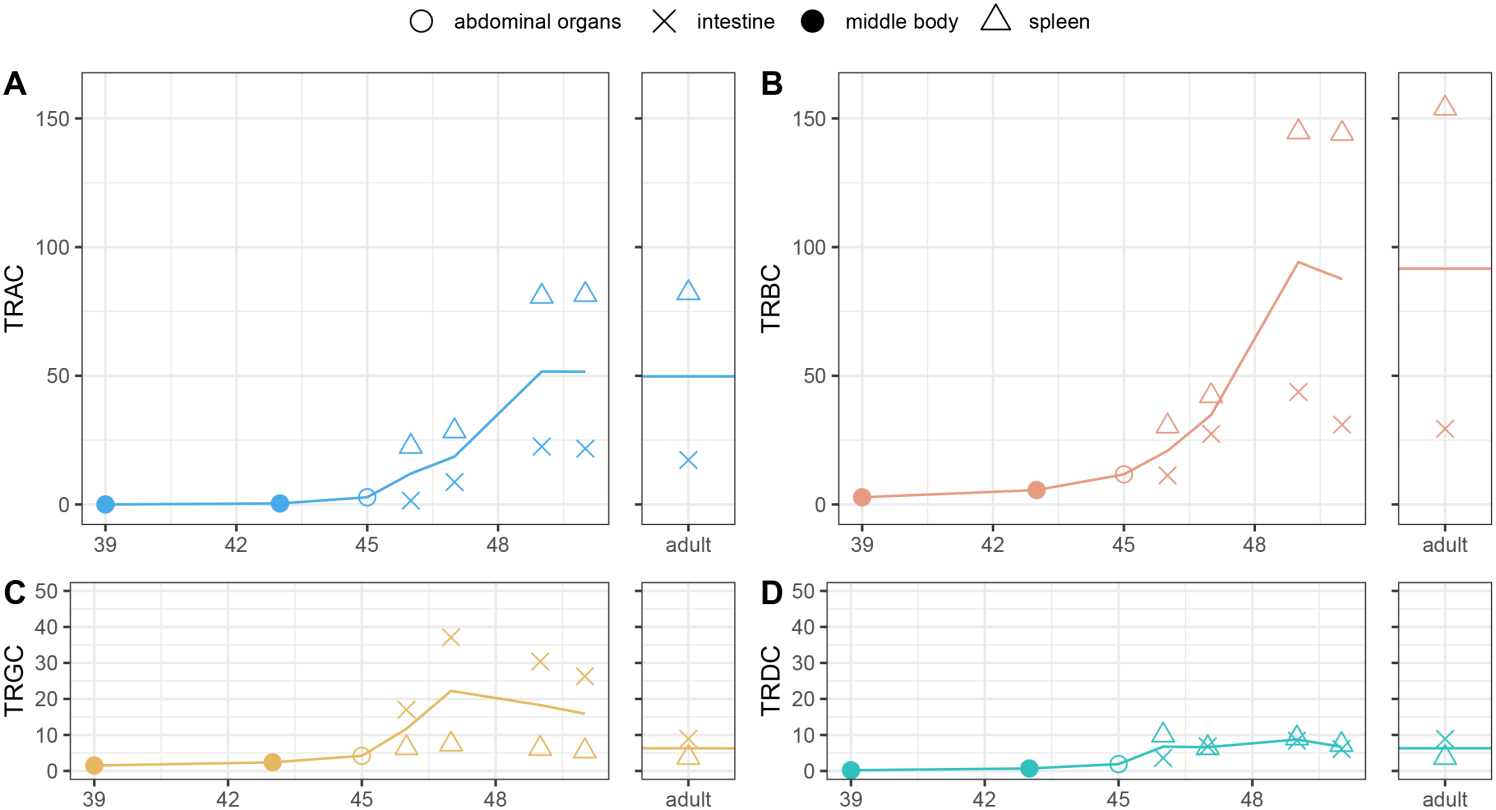
Ontogenetic TCR expression profiles. Expression levels were estimated by mapping RNA-seq reads to the constant region of each TCR chain and are shown in FPKM. Symbols represent individual tissue samples, while solid lines indicate the mean expression value per developmental stage. Developmental stages are shown on X axes and are defined according to Shi & Boucaut (49). **(A)** TRAC, **(B)** TRBC, **(C)** TRGC, **(D)** TRDC.

Overall, there is a gradual increase in the expression of all TCR chains after stage 46 (approximately 10 weeks post-fertilization and about 8 weeks post-hatching) (**Figure 6**). The earliest detectable expression appears at stage 45 for the TRB chain. Maximal expression of TRA and TRB is reached around stage 49, mid-metamorphosis, and remains at this level into adulthood. For these two chains expression in the spleen is 3–4 times higher than in the intestines, though it is clearly present in both tissues (**Figures 6A, B**). TRD shows the lowest overall expression of all chains, but follows a similar developmental trajectory (**Figure 6D**). During larval stages, TRD expression is comparable between spleen and intestine, but in adults, it becomes higher in the intestine, resembling the pattern observed for TRG. TRG expression is consistently higher in the intestines than in the spleen, peaks earlier, around stage 47 (fully developed larva), and subsequently declines (**Figure 6C**).

## 4 Discussion

Until recently, our understanding of the genomic architecture of complex, immune loci in amphibians was largely limited to anurans, especially species from the genus *Xenopus* (30, 60). In contrast, the immunogenomics of Urodela, the second-largest amphibian order, has lagged behind, primarily due to the exceptionally large genome sizes typical of this group (61). However, with recent advances in sequencing technologies, this barrier is gradually being overcome. High-quality urodele genomes have enabled the annotation of e.g., MHC in axolotl and newts (27, 62), and B and T cell receptor loci in axolotl (23, 63). Here, we contribute to this growing body of work by leveraging the recently published chromosome-scale, haplotype-resolved genome of *Pleurodeles waltl* (24) to provide the first, comprehensive description of the TCR loci in this species.

Our most intriguing finding was the separation of the TRA and TRD loci in this species, a feature confirmed through comparative analyses of available urodele genomes to be ancestral at least to newts. In all gnathostomes that possess both chains, the TRA and TRD loci are co-localized, with TRD either embedded within or positioned directly adjacent to TRA (30, 56, 64, 65). The only deviation from this pattern has been reported in a few species, where an additional, separate TRD locus exists alongside the traditional TRA/D arrangement—e.g., in Japanese flounder (66) or in Galliformes (67). The widespread retention of tight TRA-TRD linkage across vertebrates suggests a selective advantage of this arrangement, and so the consequences of separating TRA and TRD onto different chromosomes in newts remain unknown. In early studies of γδ T cells, it was hypothesized that the embedding of TRD within the TRA locus explained the mutual exclusivity of αβ and γδ TCR expression: rearrangement of the TRA locus looped out TRD genes from the genome of a developing lymphocyte, thereby precluding TRD expression in αβ T cells (68, 69). Although this view has since been challenged, α–δ and γ-β pairings are not observed in functional receptors. Whether the unique genomic organization of TCR loci in *Pleurodeles* could promote α–δ pairing remains an open question, which can be addressed with e.g., single-cell RNA sequencing.

Synteny analysis suggests that both the TRA and TRD loci in *P. waltl* have translocated away from their conserved location. In species such as *Xenopus tropicalis*, alligators, birds, and mammals, the TRA/D locus is flanked by a conserved set of genes: *METTL3*, *SALL2*, *DAD1*, and *ABHD4* (15, 30, 64, 70, 71). In *P. waltl*, this syntenic block remains intact but is devoid of TCR genes and located far from the TRD locus, yet close to the putative IgH locus. Notably, close linkage between the IgH and TRA/D loci has been reported in several species, including *Xenopus* (30), but was lost in axolotl, where TRA/D and IgH are located on opposite arms of chromosome 13 (23, 63).

Interestingly, the separation of TRA and TRD loci was observed in all other newt species with available chromosome-scale assemblies, suggesting that this genomic rearrangement was present already in the common ancestor of newts, at least ~60 million years ago (72). In contrast, reports from the axolotl, representative of the family Ambystomatidae, show that its TRD locus remains embedded within the TRA locus (23, 57). This indicates that the TRA/TRD separation must have taken place after the divergence of Ambystomatidae and Salamandridae, ~145 million years ago (72). To more precisely determine the timing of this event, genomic data from species in Salamandrinae (the sister subfamily to newts) would be essential; however, high-quality genomes are not yet available for these taxa.

TRD is considered the most evolutionarily dynamic of the TCR chains. In this study, despite some assembly uncertainties, analysis of both haplotypes revealed several notable features. These include atypical gene arrangements such as L–L–V and L–V–V units (where L stands for L-PART1 and V for V-EXON, respectively), as well as a germline-encoded, pre-joined V domain with a fixed V–J rearrangement. A comparable structure has been observed in some marsupial TCRμ chains (73); however, here the segment unit does not contain a leader peptide, and instead appears to rely on a leader sequence from an adjacent L–V cassette. Despite this, our limited repertoire analysis confirmed its expression and revealed it among the most expanded TCRδ clonotypes. Additionally, we report only the second known case, after the axolotl (12, 23), in which no D genes could be identified in the TRD locus – neither in the genome, nor in rearranged CDR3 sequences. This may thus represent a Urodela-specific feature. Notably, both TRDV and TRDJ genes are flanked by recombination signal sequences (RSS) with canonical 23-bp and 12-bp spacers, respectively, meaning direct recombination between V and J genes still complies with the 12/23 rule (74).

One key consequence of the TRD locus architecture in newts (being separated from TRA and located far from the immunoglobulin loci) is a reduced potential for TRD chain diversity. Unlike in many other species, *Pleurodeles* TRD chains cannot include TRAV genes, nor resort to Ig–TRD rearrangements involving immunoglobulin V genes (70, 75). Furthermore, the TRD locus lacks VHδ genes that enhance TRD diversity in several non-eutherian species (30, 64, 67). The absence of D genes further constrains the potential for CDR3 diversity. In addition, although the *Pleurodeles* TRG locus is organized in a fairly canonical translocon configuration, it is relatively simple, with fewer V and J genes than those observed in most other species (59, 64). Repertoire expression data—albeit limited—suggests one predominant clonotype, indicating highly constrained diversity of TRG chain. Taken together, these findings point to a highly restricted γδ T cell repertoire in *Pleurodeles*. Given consistently low expression of both TRG and TRD chains in most tissues throughout development, the role of the γδ T cell lineage is either reduced, or highly specialized and tissue-restricted (as suggested by elevated TRG expression in the intestine). Putative marginalization of the γδ T cell function may extend to other urodeles, with an even more extreme case in axolotl, where the TRG locus appears to be entirely lost (23).

The diversity and overall structure of the TRA and TRB loci in *P. waltl* are broadly comparable to those observed in other vertebrates. Notably, our analyses revealed two distinct TRA loci. Their high sequence similarity suggests a fairly recent, large *en bloc* duplication, followed by gradual divergence, with most V gene families retained on both chromosomes. Analysis of TRAC genes across six additional newt species revealed another likely case of TRA locus duplication in *Triturus* genus. However, TRAC phylogeny suggests that the duplication observed in *P. waltl* is unique to this lineage, and the one observed in *Triturus* likely represents an independent event. We note, however, that these conclusions are based only on TRAC divergence and phylogeny; a fuller picture would require analysis of entire loci (detailed characterization of which in other newt species lies beyond the scope of this study).

In general, duplications of immune receptor loci are not common. A few known examples, apart from the additional TRD loci described above, include two sets of inverted duplicated TRB loci located on two different chromosomes in salmonids (76), and two TRA/D loci in different subgenomic regions in common carp (77). These configurations likely resulted, at least partially, from retention of paralogues after whole genome duplication and allo-tetraploidization, respectively. A case of duplication and retention of a second TRA/D locus was also shown in alligators (64). Another illustrious example comes from a recent study of immunoglobulin IgH locus duplication in vespertilionid bats (78). Our initial findings in newts raise the intriguing possibility that this group may be predisposed to duplicate and retain additional TRA loci. Investigating the underlying causes (including whether the separation of TRA and TRD facilitates such events), as well as the consequences for processes such as allelic exclusion and maintenance of clonal identity will be an important direction for future research.

Repertoire sequencing confirmed expression from both TRA loci, with the vast majority of V–J pairings occurring within each locus. The instances of putative cross-locus pairing (e.g., TRA1V with TRA2J, and vice versa) may be analytical artefacts, potentially resulting from software misassignment due to genetic differences between the reference genome and that of the profiled individual, as true inter-chromosomal rearrangements are highly improbable. Nonetheless, rare inter-chromosomal insertions have been reported in B cell receptors (BCRs), where sequences derived from distant genomic regions were integrated into immunoglobulin loci during receptor assembly (79, 80). Given that BCRs and TCRs are assembled through analogous V(D)J recombination, the possibility of similar, even if rare events in T cells cannot be completely excluded and warrants further investigation.

In the TRB locus, the organization into TRBC–J–D units is conserved, with an expansion to six such units. This exceeds the numbers recorded in other vertebrates – for example, one unit in *Xenopus*, two in humans and mice, three in sheep and pig, and five in axolotl (16, 23, 30). None of the *P. waltl* genes exhibited the double C-domain structure (i.e., two Cβ-domain exons) described for axolotl TRBC 003 by (23). A large cluster of V genes is separated from the rest of the locus by a group of trypsinogen genes, a pattern observed in several other species, including humans and axolotl (16, 23). Interestingly, three smaller clusters of inverted V genes are also present, most of which show expression in repertoire sequencing data, indicating that they must undergo rearrangement by inversion. While most mammals typically have a single inverted V gene in this region (16), multiple inverted genes are more commonly seen in non-mammalian vertebrates [e.g., Chinese alligator and axolotl (23, 58)].

In addition to characterizing the genomic architecture of the TCR loci and conducting a small-scale repertoire analysis, we investigated TCR expression dynamics across ontogeny using RNA-seq data. Early histological studies in *P. waltl* examined several aspects of adaptive immunity, including thymus development and skin graft rejection. These investigations identified stages at which lymphocytes populate the thymic anlage (81) [stages 38–42 according to (82), which corresponds to 37–39 stages of (49)] and established that thymectomy by stage 52 [=45 by (49)] prevented graft rejection in adulthood, marking a critical developmental threshold for adaptive immunity (83). The marked increase in TCR expression from stage 46 (49), concurrent with MHC gene upregulation (27), supports this stage as the onset of acquired immunity. Moreover, although overall TRD expression remains low, the comparatively higher expression of TRG in intestines versus spleen is consistent with findings in many species, where γδ T cells are rare in circulating blood and lymphoid organs but abundant in the intestinal tissues (84). A similar pattern may occur in *Pleurodeles*, which may explain the difficulties encountered when amplifying the TRG repertoire from splenic RNA.

In summary, our findings reveal a unique TCR organization in newts: a complete separation of the TRA and TRD loci onto different chromosomes and a duplication of the entire TRA locus. Moreover, the combined evidence from architectural constraints, limited repertoire diversity, and low expression levels through ontogeny, suggest that γδ T cells in this species may play a highly specialized or marginal role. Beyond these insights, the immunogenetic resources established here provide a valuable foundation for future research. They open the door to comprehensive studies of TCR repertoire dynamics during development and across tissues, as well as single-cell transcriptomic approaches paired with TCR profiling—tools that will be critical for advancing our understanding of adaptive immunity in this emerging model species.

## Data Availability

The datasets presented in this study can be found in online repositories. The names of the repository/repositories and accession number(s) can be found in the article/**Supplementary Material**.
