# Supplementary material for: Unique TCR loci architecture in newts: TRA and TRD separation in Pleurodeles waltl
Source: Front Immunol. 2025 Nov 28;16:1696217. doi: 10.3389/fimmu.2025.1696217 (PMC12698447; doi:10.3389/fimmu.2025.1696217)
Supplement: Supplementary file 3 [file DataSheet3.pdf]

Supplementary Material for article:

Unique TCR Loci Architecture in Newts: TRA and TRD separation in *Pleurodeles waltl*

Magdalena Migalska[1*], Wiesław Babik[1]

[1] Institute of Environmental Sciences, Faculty of Biology, Jagiellonian University, Kraków, Poland

***Correspondence:** magda.migalska@uj.edu.pl

Table of contents

1 Supplementary Methods 2
1.1 TCR amplicon library preparation – detailed protocol 2
1.2 TCR loci annotation in the genome – additional information 2
1.3 Phylogenetic analysis – data sources 3
1.4 TCR Repertoire analysis – additional information 4
2 Supplementary Figures and Tables 5
2.1 Supplementary Figures 5
2.2 Supplementary Tables 19
3 Supplementary Data 22
4 Supplementary References 24

1 Supplementary Methods

1.1 TCR amplicon library preparation – detailed protocol

A separate reaction and library were prepared for each TCR chain. The protocol consists of three stages: 1) 5'RACE-based reverse transcription; 2) PCR 1 with *P. waltl* specific primers nested in the C region of an appropriate TCR chain; and 3) PCR 2 with indexed primers containing P5 and P7 adaptors. The species-specific primers are in Supplementary Table 1, the rest of adapters, step-out primer and other oligonucleotide sequences are available in (1).

In the first stage key components are SMARTScribe™ Reverse Transcriptase kit (TaKaRA) and a custom-made SmartNNNNa adaptor (IDT), which is a template-switch oligo containing Unique Molecular Identifier (a tag composed of 12 random nucleotides). The reaction setup consisted of two steps. First, mix 1 (250 ng of RNA and 2.5 µl of 10 µM Pw_TRX_RACE primer) was kept at 65°C for 2 min (to allow primer annealing). Next, the temperature was lowered to 42°C, and mix 2 (5× First-strand buffer, 0.3µl 20mM DTT, 1.5µl of 10µM SmartNNNNa, 1.5µl of 10mM dNTP mix, 1µl of SMARTScribe Reverse Transcriptase and 0.5µl of RNasin® Plus RNase Inhibitor, Promega) was added to each sample. Total reaction volume was 15µl. cDNA synthesis was conducted at 42°C for 1h. Afterward, products were treated with 5U USER enzyme (uracil–DNA glycosylase, NEB) for 1h at 37°C to degrade any leftover SmartNNNNa adaptor. Next, cDNA was purified using MinElute PCR purification kit (Qiagen) and eluted in 10 µl of EB.

The second stage was PCR 1. The total reaction volume was 25µl, and contained 5µl of pure cDNA, Q5® High-Fidelity 2× Master Mix (NEB), 0.5 µM of Smart20-mod step-out primer and an appropriate Pw_TRX_PCR1 reverse primer. The reverse primer, apart from a TCR C segment specific sequence, contained an overhang with partial Illumina adapter/sequencing primer. The PCR conditions were: 98°C for 1 min, followed by 23 cycles of 98°C for 10s, 65°C for 20s, 72°C for 40s, and a final extension at 72°C for 4 min. PCR 1 products were purified using AMPure XP beads (Beckman Coulter) with 1:0.6 DNA-to-beads ratio. DNA was eluted in 20µl of nuclease-free H_2O.

The third stage was PCR 2. The total reaction volume was 20µl, and contained the following: 5µl of the purified product of PCR 1 used as a template, Q5® High-Fidelity 2× Master Mix (NEB), a unique combination of P5_50X and P7_70X primers (1.25µM each), which contained indices that would be used for de-multiplexing of samples pooled in a sequencing run as well as overhangs serving as adapter for Illumina sequencing instruments. The PCR cycling conditions were as described for PCR 1, but with 12 cycles and 5min of final elongation. The products of PCR 2 were run on 1.5% agarose gel immediately after reaction. Bands of desired size (TCR α, β, γ ~650; TCR δ ~700-800 bp) were purified using ZymoClean Gel DNA Recovery Kit (Zymo research). The DNA was eluted in 15µl of warm EB and its concentration was measured using HS dsDNA Qubit kit (ThermoFisher Scientific).

1.2 TCR loci annotation in the genome – additional information

Mapping partial TCR amplicons to the genome

One of the steps in the annotation protocol (see Main Text) involved mapping partial amplicon reads to genomic fragments containing putative TCR loci. Due to the library preparation method, forward reads of the TCR amplicons always contained the 5′ portion of the transcript, including the 5′UTR,

leader peptide, and part of the V segment. These were therefore used to assist in detecting leader peptide–containing exons of each L–V–GENE–UNIT. Prior to mapping, UMIs and 5′RACE adapter sequences were removed from the reads using `cutadapt` (2) (with the argument: `-g GGTATCAACGCAGAGTNNNNNNNNNNNNNNNNCTTGGGG`. Next, amplicons were subsampled to a maximum of 50,000 reads (to accelerate mapping) and aligned to the reference genome using `minimap2` (3). The resulting mappings were visualized in IGV (4) alongside putative V segments automatically extracted by *VJ-gene-finder* v2.0 (5). Coordinates of the putative L-PART1 exons were exported in BED format and subsequently used to aid manual annotation of the TCR loci.

1.3 Phylogenetic analysis – data sources

For the TRA and TRB V segment analyses, we selected a single representative sequence per V gene family, with families defined as sharing ≥75% nucleotide sequence identity. Where possible, we used the first described locus and allele as the representative. In cases lacking subfamily classification, sequence similarity was assessed, and one sequence was selected arbitrarily. For TRBC segment analysis, only the Constant domain corresponding to the extracellular immunoglobulin-like domain (exon 1 of the C gene) was used. All sequences used in these analyses are available at https://osf.io/f6kez/ see section Supplementary Data).

Sequence Sources by Species:

- **Zebrafish (*Danio rerio*):**

TRAV: Seelye et al., *Immunogenetics* (2016) (6)
TRBV, TRBC: Meeker et al., *Immunogenetics* (2010) (7)
TRDV: retrieved from IMGT/GENE-DB, version 3.1.42
TRGV: Crider et al., *Front Immunol* (2021) (8)

- **Axolotl (*Ambystoma mexicanum*):**

All segments from Pacheco-Olvera et al., *Front Immunol* (2025) (9)

- **Chicken (*Gallus gallus*):**

All segments from Früh et al., *Front Immunol* (2024) (5)

- **Human (*Homo sapiens*):**

All TCR segments retrieved from IMGT/GENE-DB, Giudicelli et al., *Nucleic Acids Res* (2005) (10) version 3.1.42.)

- ***Xenopus laevis:***

TRBC – partial BC170535.1

1.4 TCR Repertoire analysis – additional information

Despite the overall high quality of sequencing reads, initial inspection revealed substantial variation in the proportion of target reads (i.e., containing both V and J segments of the desired chain). This issue was most pronounced for TRG, where up to 90% of reads were non-target. Amplification problems for TRG could not be resolved despite redesigning and optimizing primers, and are likely attributable to its low expression level in the interrogated tissue (spleen). To mitigate the impact of non-target reads, amplicons were prefiltered by retaining only reads that matched the reference V segment sequences with a BLAST e-value threshold of 1e-10. The filtered reads were then analyzed with MiXCR v4.7.0 (local Conda installation) (11).

First, for each TCR locus, a custom MiXCR reference library was assembled using the `buildLibrary` option, specifying `--v-gene-feature` as "VRegion" and providing the appropriate V-REGION, J-EXON, D-EXON, and first exon(s) of C-REGION sequences in FASTA format. For TRD, only the haplotype 2 annotation was used, as multiple lines of evidence suggested it more accurately reflected the true locus structure. In most cases, MiXCR failed to automatically identify the required `CDR3Begin` and `FR4Begin` features in the supplied V and J gene sequences. These features were necessary for basic repertoire analysis, therefore were added manually to the library JSON file or via custom Python scripts, based on position inferred from an aligned, multi-FASTA file.

Next, repertoire of each chain was analyzed separately, using `mixcr analyze` command, which executes a complete analysis pipeline from the raw FASTQ files to clonotype tables. The analyses were run with a *`generic-amplicon-with-umi`* preset, and additional options: `--rna, --tag-pattern "^N{0:6}aagcagtggtatcaacgcagag(UMI:TNNNNTNNNNTNNNNT)CTTgggg(R1:*)\^N{0:6}(R2:*)", --rigid-left-alignment-boundary, --floating-right-alignment-boundary C, --assemble-clonotypes-by CDR3.`

Subsequently each amplicon was processed with the MiXCR command `mixcr postanalysis individual`, which computed repertoire characteristics for each of the clonesets separately. Read counts were downsampled to 400,000, clonotype abundances were weighted by UMI counts, and only productive rearrangements were retained (used options: `--default-downsampling count-read-fixed-400000 --default-weight-function umi --only-productive.`)

2 Supplementary Figures and Tables

2.1 Supplementary Figures

TRA1 sc3b

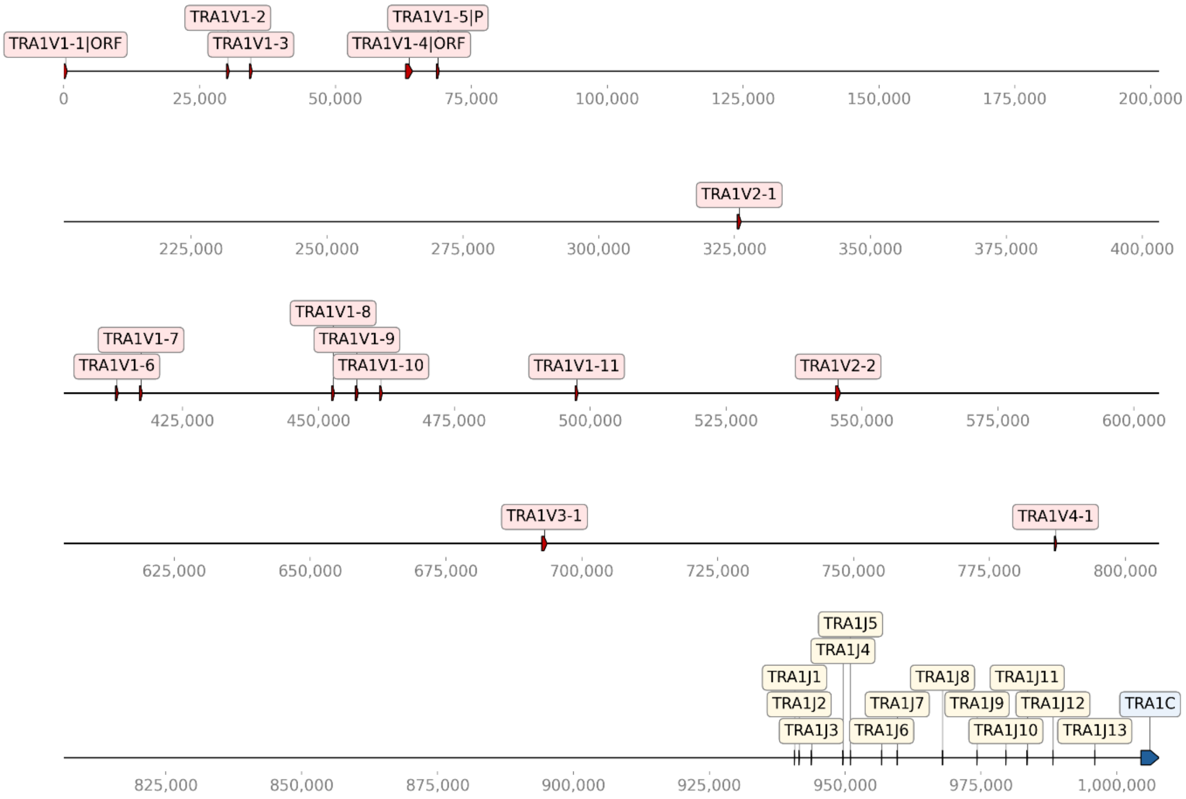

TRA2 sc4a

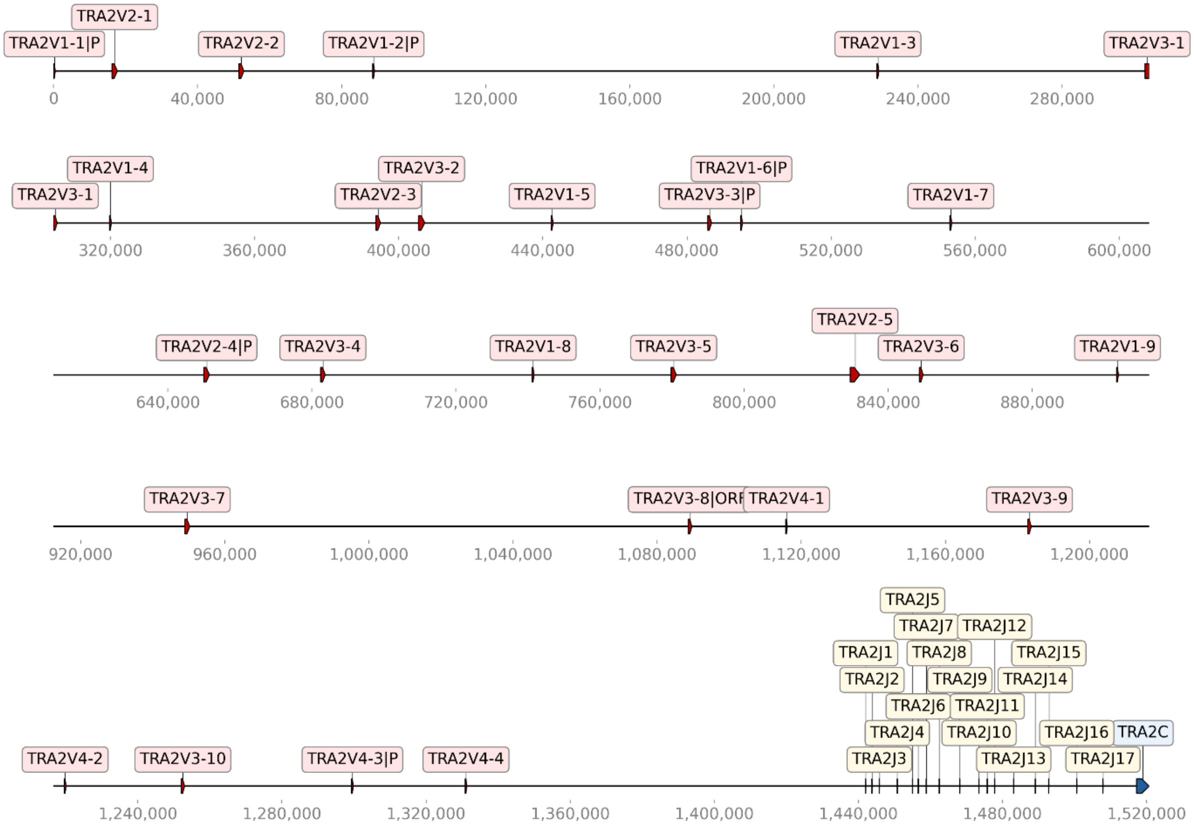

Supplementary Figure 1. The TCR α loci (TRA1 and TRA2) of *Pleurodeles waltl* on scaffolds 3b and 4a. The gene map is drawn to scale, in the transcriptional orientation of the locus, as inferred from the orientation of the C genes. Coordinates are numbered relative to the first annotated feature on each locus. Position 0 on TRA1 corresponds to position 221,009,097on scaffold 3b, and the last position is 220,001,322 on the same scaffold. Position 0 on TRA2 corresponds to position 2,849,870on scaffold 4a, and the last position is 1,329,156 on the same scaffold. Labels indicate the locus and segment type—V (variable; the entire V-gene cassette, including L-PART1 and V-EXON), J (joining), or C (constant; all exons). All gene segments are functional unless marked otherwise; suffix "|P" indicates a pseudogene, and "|ORF" denotes an open reading frame (see main text). Arrows show transcriptional orientation; all J genes are in the same orientation as the locus.

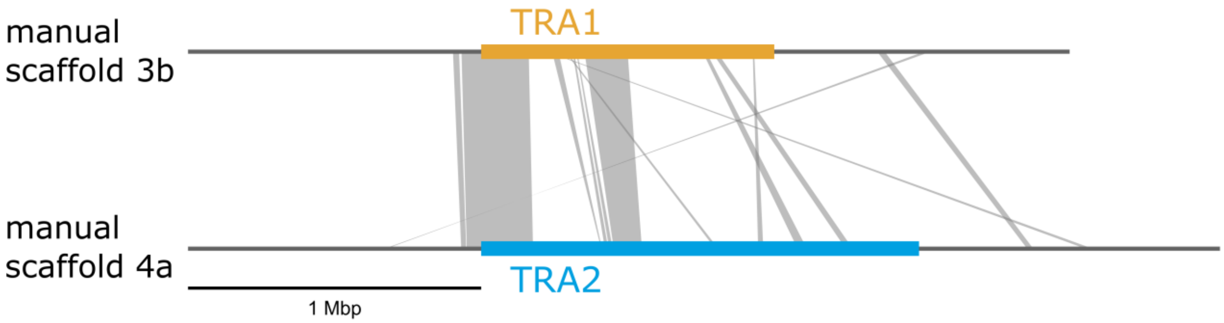

Supplementary Figure 2. Synteny blocks (minimum length 10 kb) identified between the TRA1 and TRA2 loci, including 1 Mb of flanking sequence on each side. The full displayed regions correspond to positions 219,001,322-222,009,097 on Scaffold 3b and 329,156-3,849,870 on Scaffold 4a; regions encompassed by each TRA locus are highlighted with colored bars.

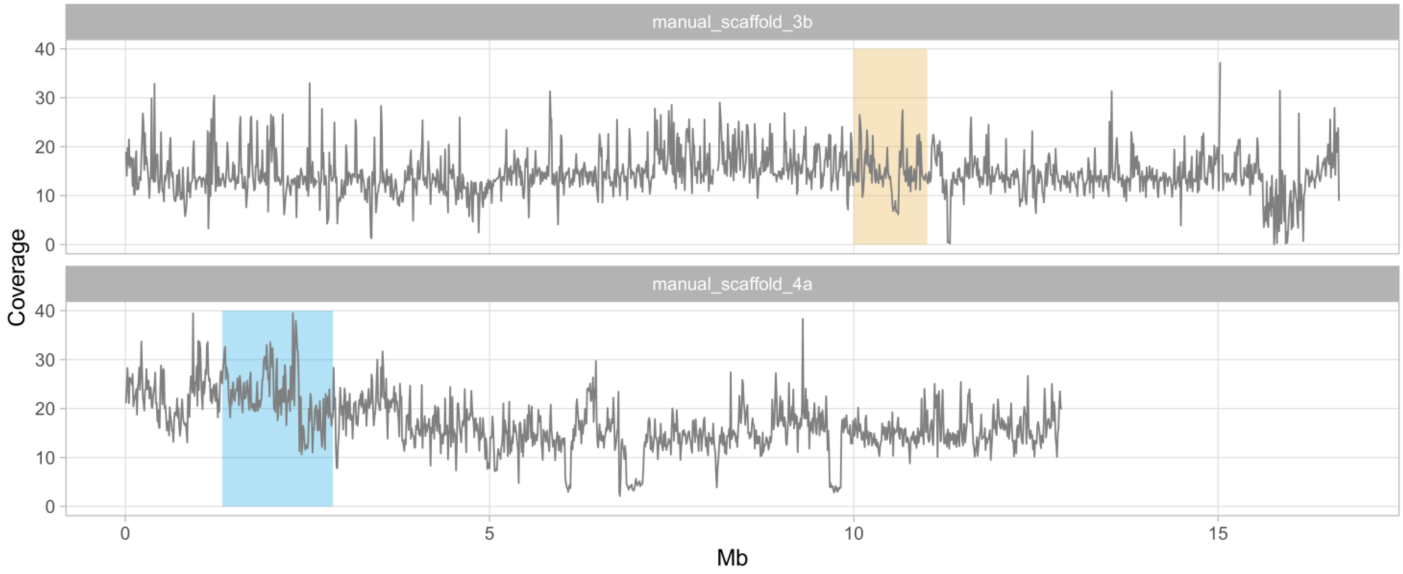

Supplementary Figure 3. Read coverage across genomic regions encompassing the two TRA loci, extended to include up to 10 Mb of surrounding context or to the end of each scaffold (corresponding to the chromosome end). The displayed regions correspond to positions 210,001,322-226,660,143 on Scaffold 3b and 1-12,849,870 on Scaffold 4a. Regions corresponding to each TRA locus are highlighted with colored shading (yellow for TRA1 and blue for TRA2).

Haplotype 1 TRD sc6

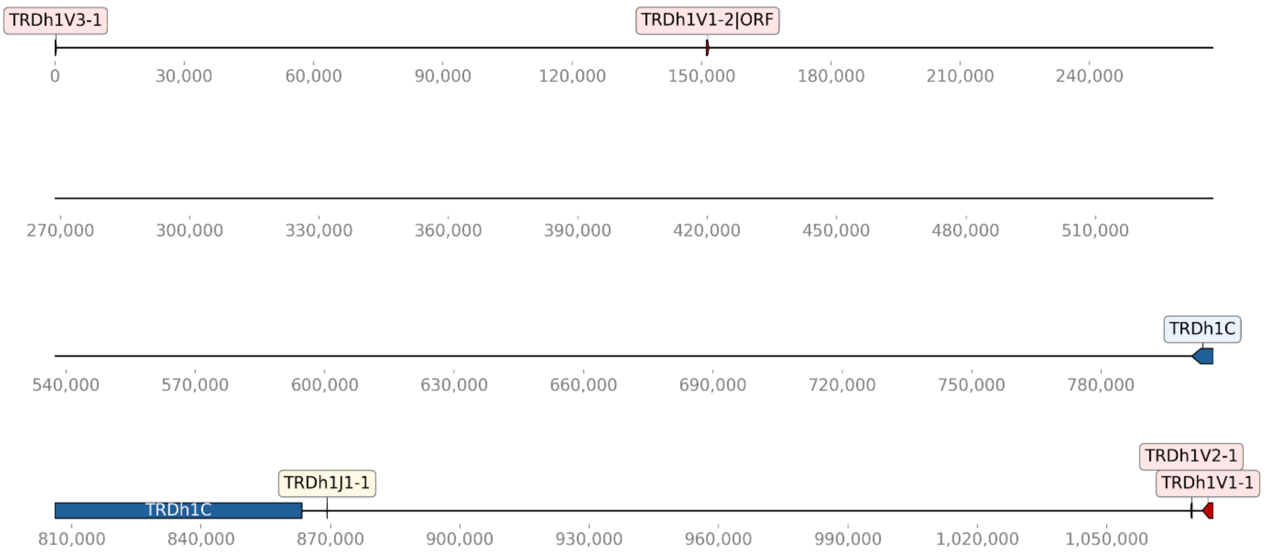

Haplotype 2 TRD sc6

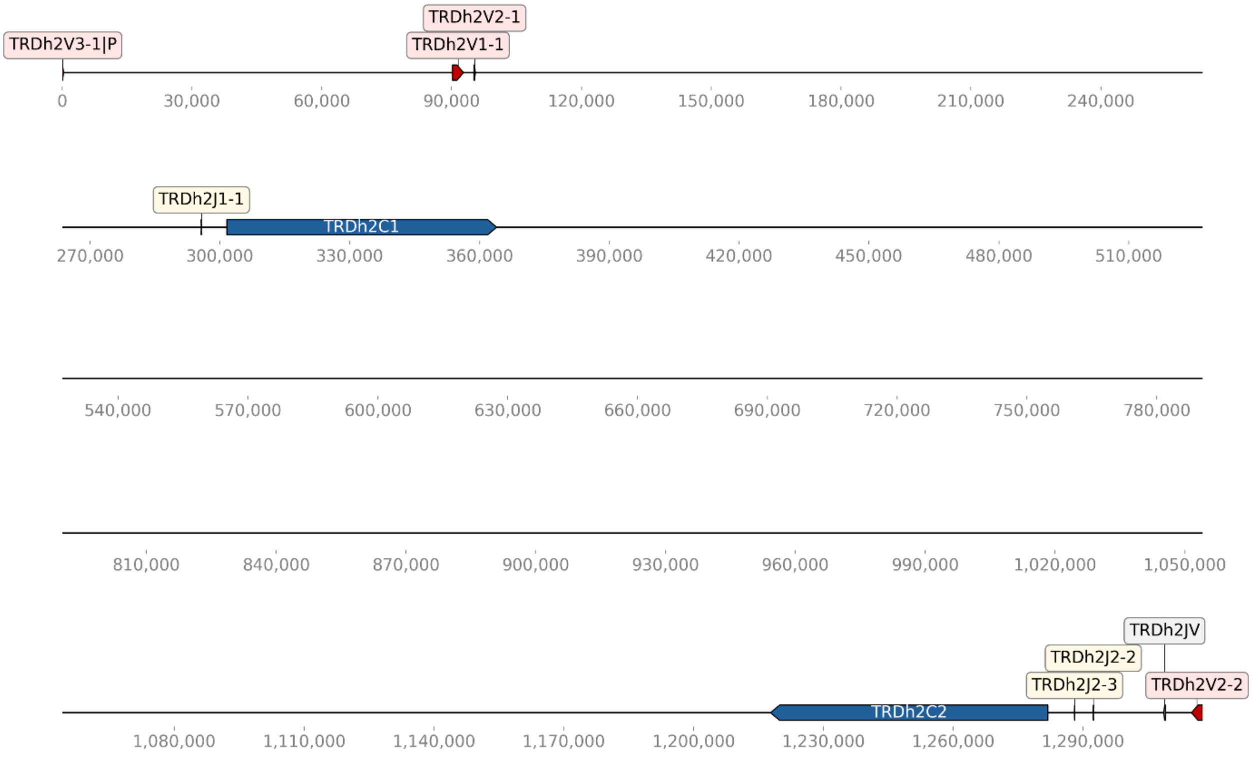

Supplementary Figure 4. The TCR δ locus of *Pleurodeles waltl* on scaffold 6 of haplotype 1 and 2 (TRDh1 and TRDh2, respectively). The gene map is drawn to scale, with coordinates numbered relative to the first annotated feature of each locus. Given the uncertainty regarding the true structure and orientation of the locus, the figure follows the scaffold orientation. Position 0 on TRDh1 corresponds to position 682,022,177; position 0 on TRDh2 corresponds to 1,043,651,231. Labels indicate the locus and segment type—V (variable), J (joining), or C (constant; all exons). All gene segments are functional unless marked otherwise: "ORF" denotes an open reading frame, and "P" indicates a pseudogene. Arrows show transcriptional orientation; single J segment at TRDh1 is in reverse orientation, in case of TRDh2 all but one (TRDh2J1-1) are in reverse orientation. Segment TRDh2JV (grey label) indicates germline-joint segment.

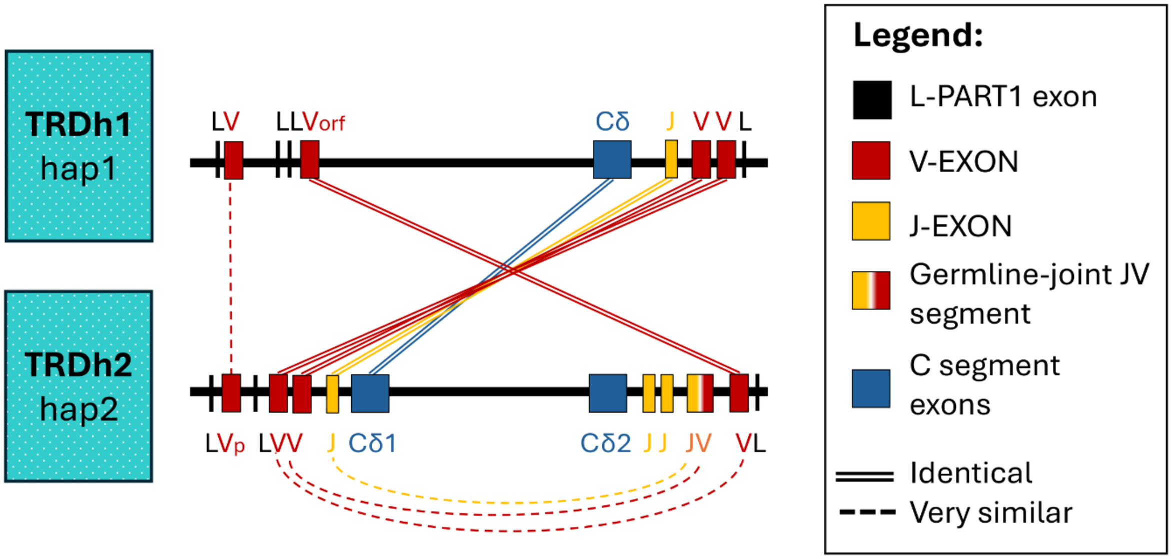

Supplementary Figure 5. ***Pleurodeles waltl*** **TRD loci – schematic representation of correspondence between haplotypes.** Identical segments are connected by double solid lines, while similar segments are connected by dashed lines. A legend for gene segment groups is provided. Drawn not to scale. Based on annotation of a reference haplotype 1 (hap1) - aPleWal.hap1.mc02.scaffolds.fa.gz at (14), available also as NCBI RefSeq GCF_031143425.1 (aPleWal1.hap1.2022112); and an alternative haplotype 2 (hap2) - aPleWal.hap2.mc02.scaffolds.fa.gz at (14), available also as GenBank assembly GCA_031142525.1 (aPleWal1.hap2.20221129).

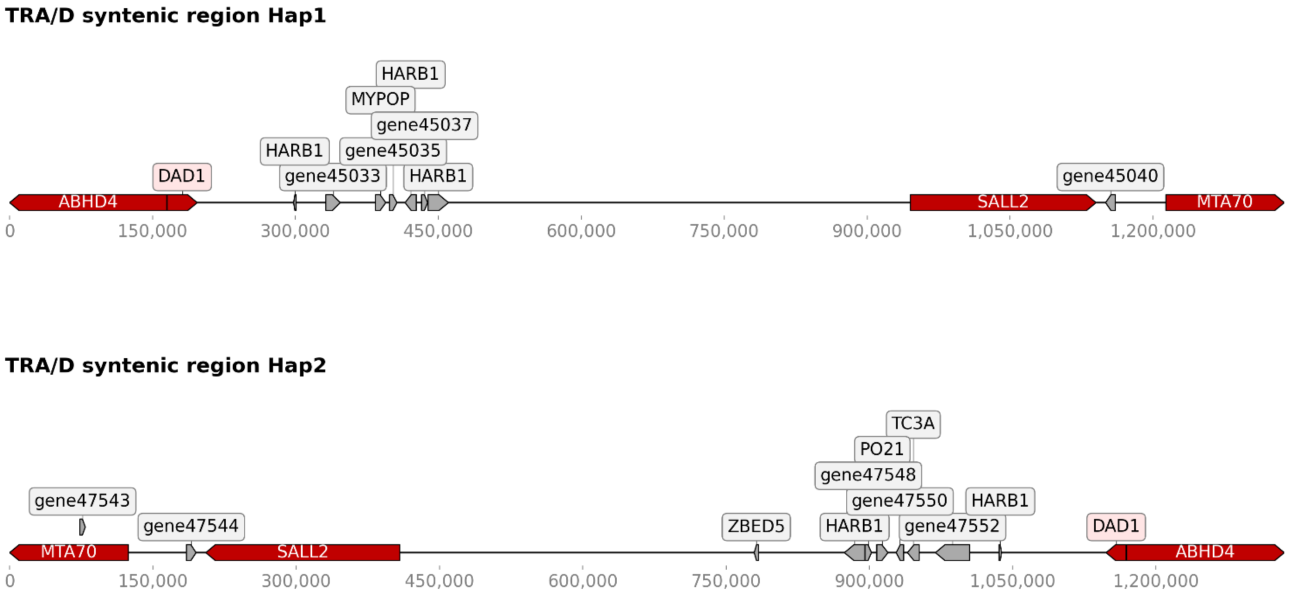

Supplementary Figure 6. TRA/D syntenic region in *Pleurodeles waltl.* The interval (< 750 kb) between genes typically flanking the TRA/D locus in many vertebrates contains no annotated TRA or TRD genes. Genes of conserved synteny are shown in red: *ABHD4*, abhydrolase domain-containing protein 4; *DAD1*, defender against cell death 1; *MTA70=METTL3*, methyltransferase-like 3; *SALL2*, Sal-like protein 2. Other annotated genes in the region include: TC3A, transposable element Tc3 transposase; *HARB1*, putative nuclease HARBI1; *MYPOP*, Myb-related transcription factor, partner of profilin; PO21, retrovirus-related Pol polyprotein from a type-1 retrotransposable element; *ZBED5*, zinc finger BED domain-containing protein 5. For haplotype 1 (Hap1), the 0 coordinate corresponds to position 362,481,072 of scaffold 6; for haplotype 2 (Hap2), it corresponds to position 1,366,098,803 of scaffold 6.

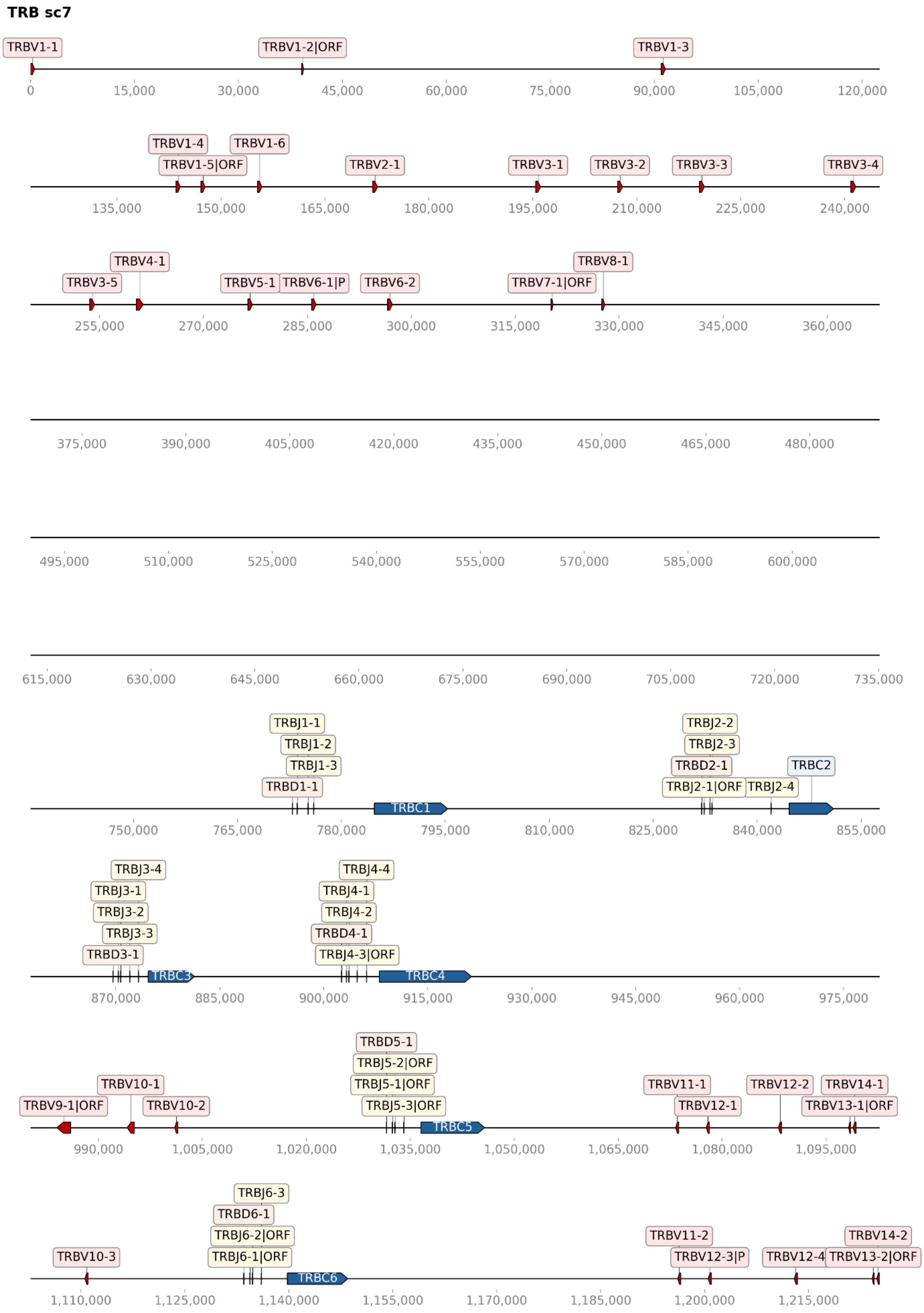
TRB sc7
TRBV1-1
TRBV1-2|ORF
TRBV1-3
TRBV1-4
TRBV1-6
TRBV1-5|ORF
TRBV2-1
TRBV3-1
TRBV3-2
TRBV3-3
TRBV3-4
TRBV4-1
TRBV3-5
TRBV5-1
TRBV6-1|P
TRBV6-2
TRBV8-1
TRBV7-1|ORF
TRBJ1-1
TRBJ1-2
TRBJ1-3
TRBD1-1
TRBC1
TRBJ2-2
TRBJ2-3
TRBD2-1
TRBC2
TRBJ2-1|ORF
TRBJ2-4
TRBJ3-4
TRBJ3-1
TRBJ3-2
TRBJ3-3
TRBD3-1
TRBC3
TRBJ4-4
TRBJ4-1
TRBJ4-2
TRBD4-1
TRBJ4-3|ORF
TRBC4
TRBD5-1
TRBJ5-2|ORF
TRBJ5-1|ORF
TRBJ5-3|ORF
TRBC5
TRBV10-1
TRBV9-1|ORF
TRBV10-2
TRBV11-1
TRBV12-2
TRBV14-1
TRBV12-1
TRBV13-1|ORF
TRBJ6-3
TRBD6-1
TRBJ6-2|ORF
TRBJ6-1|ORF
TRBC6
TRBV10-3
TRBV11-2
TRBV14-2
TRBV12-3|P
TRBV12-4
TRBV13-2|ORF

Supplementary Figure 7. The TCR β locus (TRB) of *Pleurodeles waltl* on scaffold 7. The gene map is drawn to scale in the transcriptional orientation of the locus, as inferred from the orientation of the C genes. This orientation is opposite to that of the scaffold. Coordinates numbered relative to the first annotated feature: position 0 corresponds to position 19,122,406on scaffold 7, last position is 17,897,156. Labels indicate the locus and segment type— V (variable; the entire V-gene cassette, including L-PART1 and V-EXON), D (diversity), J (joining), or C (constant; all exons). All gene segments are functional unless marked otherwise; suffix "|P" indicates a pseudogene, and "|ORF" denotes an open reading frame (see main text). Arrows show transcriptional orientation; all J and D segments are in the same orientation as the locus.

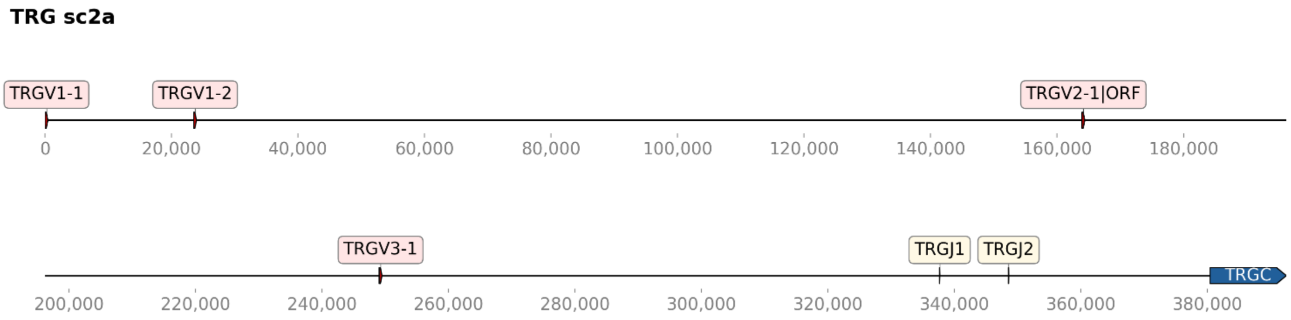

Supplementary Figure 8. The TCR γ locus (TRG) of *Pleurodeles waltl* on scaffold 2a. The gene map is drawn to scale, in the transcriptional orientation of the locus, as inferred from the orientation of the C genes. This orientation is opposite to that of the scaffold. Coordinates are numbered relative to the first annotated feature; position 0 corresponds to position 612,337,084 on scaffold 2b, last position is 611,944,613. Labels indicate the locus and segment type—V (variable, the entire V-gene cassette, including L-PART1 and V-EXON), J (joining), or C (constant; all exons). All gene segments are functional unless marked otherwise; "|ORF" denotes an open reading frame (see main text). Arrows show transcriptional orientation; all segments are in reverse orientation.

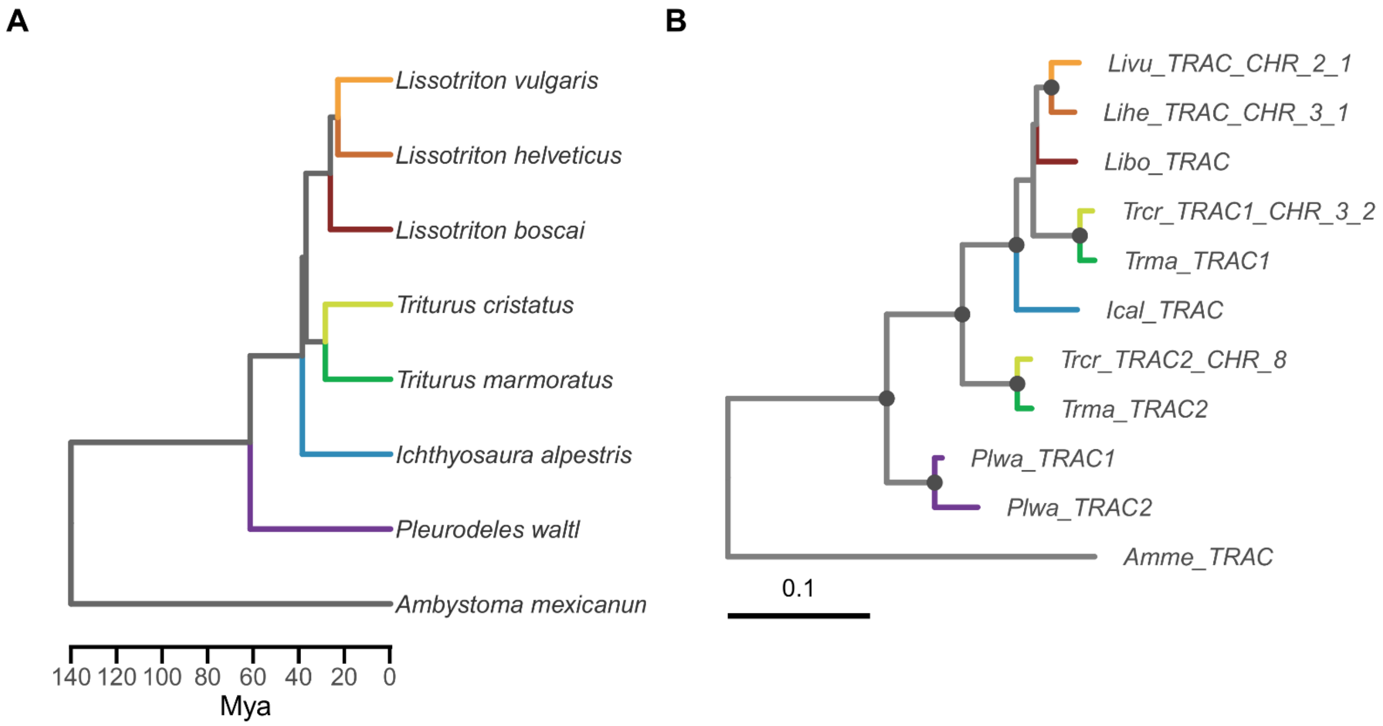

Supplementary Figure 9. Phylogeny of seven newt species (A) and Maximum Likelihood phylogeny of newt TRAC segments (B). (A) Time-calibrated phylogeny of the studied species (12). (B). Dots indicate clades with bootstrap support of min. 70%. Species names are abbreviated using the first two letters of the genus and species epithet (e.g., *Pleurodeles waltl* = Plwa). Branches are color-coded according to species.

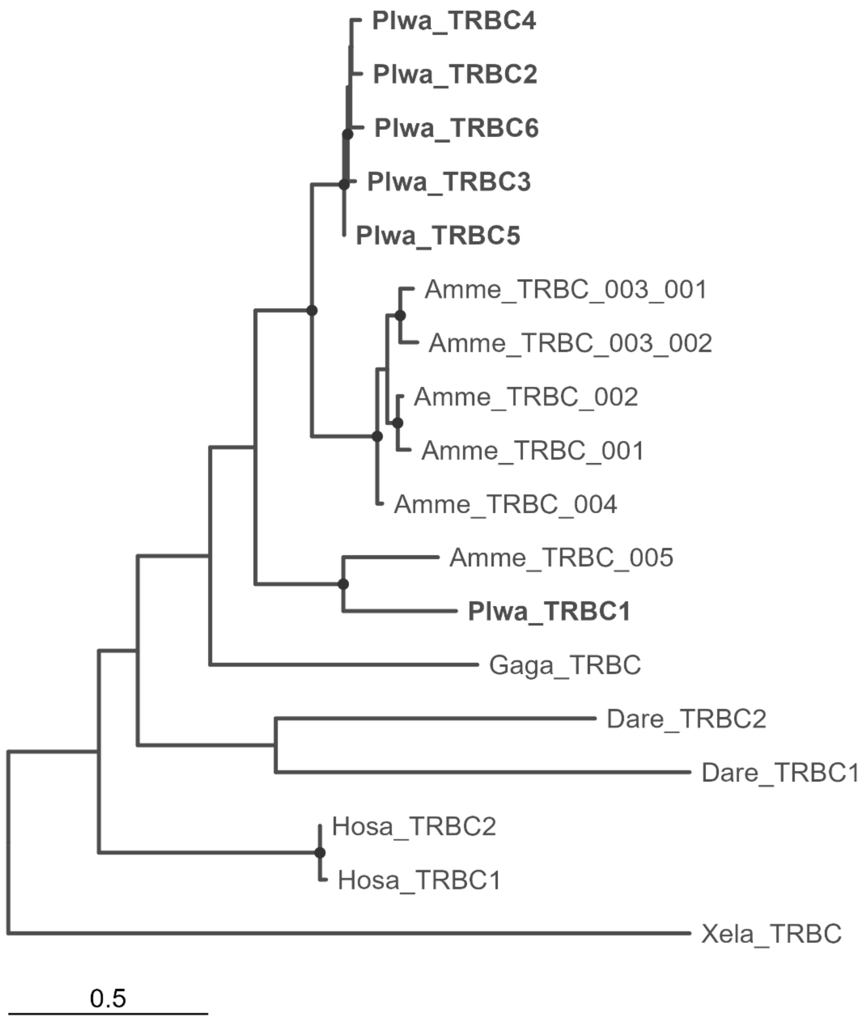

Supplementary Figure 10. Maximum Likelihood phylogeny of vertebrate TRB constant segments. Only Constant domain (EX1). Dots indicate clades with bootstrap support of min. 70%. Species abbreviations: Hosa – *Homo sapiens*; Gaga – *Gallus gallus*; Amme – *Ambystoma mexicanum*; Plwa – *Pleurodeles waltl*; Xela – *Xenopus leavis*; Dare – *Danio rerio*.

2.2 Supplementary Tables

Supplementary Table 1. Species-specific primers used for TCR repertoire library preparation.

Step	TCR chain	Name	5'->3' Sequence	notes
5'RACE	TCR α	Pw_TRA_RACE	gggagaagtctgtgatgagg	
5'RACE	TCR β	Pw_TRBc3_RACE	catggtcagggtagaacttg	mixed in 3:1 c3:c4 ratio‡
5'RACE	TCR β	Pw_TRBc4_RACE	cagaagggttgaacagcac	
5'RACE	TCR δ	Pw_TRD_RACE	tctgccgctggtggaattcatg	
5'RACE	TCR γ	Pw_TRG_RACE	ggcataagtacgtcagcttc	
PCR 1	TCR α	Pw_TRA_PCR1	†[overhang]ttaggaggtagactgaaggtgg	
PCR 1	TCR β	Pw_TRBc3_PCR1	†[overhang]aagagccacgtttggttc	mixed in 3:1 c3:c4 ratio‡
PCR 1	TCR β	Pw_TRBc4_PCR1	†[overhang]ctctgactggtacatcttc	
PCR 1	TCR δ	Pw_TRD_PCR1	†[overhang]gatggctgcaawtttgtttg	
PCR 1	TCR γ	Pw_TRG_PCR1	†[overhang]agaaggaggcagtatttttgc	

†overhang sequence: GTCTCGTGGGCTCGGAGATGTGTATAAGAGACAG

‡ Primers for the TCRβ chain – primers with suffix *c3* are complementary to *Pleurodeles* segments TRBC2–C6, while primers *c4* to TRBC1. The naming was based on similarity to axolotl sequences described by Fellah et al. (2002).

Supplementary Table 2. Repertoire diversity measures reported by MiXCR. During analysis read counts were downsampled to 400,000; clonotype abundances were weighted by UMI counts; only productive rearrangements were retained. Detailed explanations and references for the diversity metrics are available at: https://mixcr.com/mixcr/reference/mixcr-postanalysis/#diversity-measures.

**Chain**	**TRA**	**TRB**	**TRG**	**TRD**
Total amplicon reads	1,476,737	1,777,501	2,593,764	537,097
Pre-filtered reads*	1,434,041	1,707,795	462,439	411,231
Observed diversity	4207	8869	49	134
Shannon-Wiener diversity	2879	7844	31	19
Normalized Shannon-Wiener index	0.95	0.99	0.88	0.60
Inverse Simpson index	1340	5607	18	7
Gini index	1.00	1.00	1.06	1.14
Chao1 estimate	11495	37765	234	248
Efron-Thisted estimate	16911	56813	88	266
d50	772	1381	5	2

*see section: *TCR Repertoire analysis – additional information*

Supplementary Table 3. Samples used for ontogenetic TCR expression profiling. Sequencing reads are available at the European Nucleotide Archive under ENA Research Project accession PRJEB90989. Developmental stages follow Shi & Boucaut (13), with age indicated in weeks post-fertilization.

ENA Sample Accession	Sample ID	stage	tissue	age [weeks]
SAMEA118593042	Pw_25200_2	39	middle body part	4.5
SAMEA118593045	Pw_25215_2	43	middle body part	6
SAMEA118593050	Pw_25219_4	45	abdominal organs	8
SAMEA118593054	Pw_25231_4	46	intestine	10
SAMEA118593055	Pw_25231_5	46	spleen	10
SAMEA118593066	Pw_25463_4	47	intestine	15
SAMEA118593067	Pw_25463_5	47	spleen	15
SAMEA118593069	Pw_25487_4	49	intestine	19
SAMEA118593070	Pw_25487_5	49	spleen	19
SAMEA118593072	Pw_25488_4	50	intestine	19
SAMEA118593073	Pw_25488_5	50	spleen	19
SAMEA118593230	Pw_25183_5	adult	spleen	NA
SAMEA118593231	Pw_25183_6	adult	intestine	NA

3 Supplementary Data

Supplementary Materials, apart from this file, contain the following files:

- *Hap1_TR_annotation.gff* – annotation file with genomic location of all TRA/B/G/D features on haplotype 1 (aPleWal.hap1.mc02.scaffolds.fa.gz at (14))
- *Hap2_TRD_annotations.gff* – annotation file with genomic location of all TRD features on haplotype 2 (aPleWal.hap2.mc02.scaffolds.fa.gz at (14))

Additional files are available in Center for Open Science repository https://osf.io/f6kez/

Folder D1 – TCR loci annotation

- *Hap1.allFeatures.fasta* – Sequences of all annotated features in haplotype 1
- *Hap2.TRDFeatures.fasta* – Sequences of annotated TRD features in haplotype 2

Folder D2 – Phylogenetic analyses

- *TRAV_tree.fasta* – sequences used to generate TRAV phylogenetic trees
- *TRAV_tree_metadata.tsv* – inclusion of sequences in Fig 4A (species) and Fig 2B (urodela)
- *TRAC_tree.fasta* - sequences used to generate TRAC phylogenetic tree (Supplementary Fig 9)
- *TRBC_tree.fasta* - sequences used to generate TRBC phylogenetic tree (Supplementary Fig 10)
- *TRBV_tree.fasta* – sequences used to generate TRBV phylogenetic trees
- *TRBV_tree_metadata.tsv* – inclusion of sequences in Fig 4B (species) and Fig 3B (urodela)
- *TRDV_tree.fasta* - sequences used to generate TRDV phylogenetic tree (Fig 4C).
- *TRGV_tree.fasta* - sequences used to generate TRGV phylogenetic tree (Fig 4D).

Folder D3 - MiXCR custom library files

V and J reference files:

- *Hap1_J-EXON_TRA.fasta*
- *Hap1_J-EXON_TRB.fasta*
- *Hap1_J-EXON_TRG.fasta*
- *Hap1_V-REGION_TRA.fasta*
- *Hap1_V-REGION_TRB.fasta*
- *Hap1_V-REGION_TRG.fasta*
- *Hap2_J-EXON_TRD.fasta*
- *Hap2_V-REGION_TRD.fasta*

JSON custom libraries:

- *upd_Pleuro_TRA.json*
- *upd_Pleuro_TRB.json*

- *upd_Pleuro_TRDh2.json*
- *upd_Pleuro_TRG.json*

4 Supplementary References

1. Migalska M, Sebastian A, Radwan J. Profiling of the TCRβ repertoire in non-model species using high-throughput sequencing. *Sci Rep* (2018) 8:11613. doi: 10.1038/s41598-018-30037-0

2. Martin M. Cutadapt removes adapter sequences from high-throughput sequencing reads. *EMBnet.journal* (2011) 17:10–12. doi: 10.14806/EJ.17.1.200

3. Li H. Minimap2: pairwise alignment for nucleotide sequences. *Bioinformatics* (2018) 34:3094–3100. doi: 10.1093/bioinformatics/bty191

4. Robinson JT, Thorvaldsdóttir H, Winckler W, Guttman M, Lander ES, Getz G, Mesirov JP. Integrative genomics viewer. *Nat Biotechnol* (2011) 29:24–26. doi: 10.1038/nbt.1754

5. Früh SP, Früh MA, Kaufer BB, Göbel TW. Unraveling the chicken T cell repertoire with enhanced genome annotation. *Front Immunol* (2024) 15:1–19. doi: 10.3389/fimmu.2024.1359169

6. Seelye SL, Chen PL, Deiss TC, Criscitiello MF. Genomic organization of the zebrafish (Danio rerio) T cell receptor alpha/delta locus and analysis of expressed products. *Immunogenetics* (2016) 68:365–379. doi: 10.1007/S00251-016-0904-3,

7. Meeker ND, Smith ACH, Frazer JK, Bradley DF, Rudner LA, Love C, Trede NS. Characterization of the zebrafish T cell receptor β locus. *Immunogenetics* (2010) 62:23–29. doi: 10.1007/S00251-009-0407-6/FIGURES/9

8. Crider J, Quiniou SMA, Felch KL, Showmaker K, Bengtén E, Wilson M. A Comprehensive Annotation of the Channel Catfish (Ictalurus punctatus) T Cell Receptor Alpha/Delta, Beta, and Gamma Loci. *Front Immunol* (2021) 12:786402. doi: 10.3389/FIMMU.2021.786402/BIBTEX

9. Pacheco-Olvera D, Saint Remy-Hernández S, Godoy-Lozano E, Téllez-Sosa J, Valdovinos-Torres H, Curiel-Quesada E, López-Macías C, Martínez-Barnetche J. Genomic characterization of the T-cell receptor loci in Ambystoma mexicanum. *Front Immunol* (2025) Volume 16: doi: 10.3389/fimmu.2025.1656386

10. Giudicelli V, Chaume D, Lefranc MP. IMGT/GENE-DB: a comprehensive database for human and mouse immunoglobulin and T cell receptor genes. *Nucleic Acids Res* (2005) 33: doi: 10.1093/NAR/GKI010

11. Bolotin DA, Poslavsky S, Mitrophanov I, Shugay M, Mamedov IZ, Putintseva E V, Chudakov DM. MiXCR: software for comprehensive adaptive immunity profiling. *Nat Methods* (2015) 12:380–381. doi: 10.1038/nmeth.3364

12. Stewart AA, Wiens JJ. A time-calibrated salamander phylogeny including 765 species and 503 genes. *Mol Phylogenet Evol* (2025) 204: doi: 10.1016/j.ympev.2024.108272

13. Shi DL, Boucaut JC. The chronological development of the urodele amphibian Pleurodeles waltl (Michah). *Int J Dev Biol* (1995) 39:427–441. doi: 10.1387/IJDB.7577434

14. Brown T. Pleurodeles waltl genome assembly. V4 ed. Edmond (2022). doi: doi:10.17617/3.90C1ND
